# Supplementary material for: A regulatory module mediating temperature control of cell-cell communication facilitates tree bud dormancy release
Source: EMBO J. 2024 Oct 3;43(23):4. doi: 10.1038/s44318-024-00256-5 (PMC11612439; doi:10.1038/s44318-024-00256-5)
Supplement: Supplementary file 1 — Appendix [file 44318_2024_256_MOESM1_ESM.pdf]

Appendix

A regulatory module mediating temperature control of cell-cell communication facilitates tree bud dormancy release

Shashank K. Pandey et al.

Table of Contents:

Appendix Figure S1 2

Appendix Figure S2 4

Appendix Figure S3 5

Appendix Figure S4 6

Appendix Figure S5 7

Appendix Figure S6 8

Appendix Method 10

Appendix Table S1 11

Appendix Table S2 12

Appendix References 13

A

Potri.001G328600/ Potra002319g17692/ Potrx027149g00010

MVRGKVQLQRIEDKSSRQVCFSKRKRGLLKKAKELSVLCDVEMAVIIFSSTGKLFEFCSG
NSLRNILERYDTHKTKSQEIAICKNVDKTKQNHHAEYMGSSYMDANPLQMVQRYFEGKNI
EQLNITQLMQLERELDSTLLYTRGRKTEAMMKSVTALHQKEQDLTDENNLIEREISAIINNG
NLAGQHGRVVEDPDCVHPSPLDLFHF

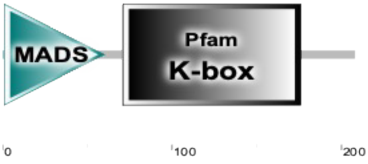

B

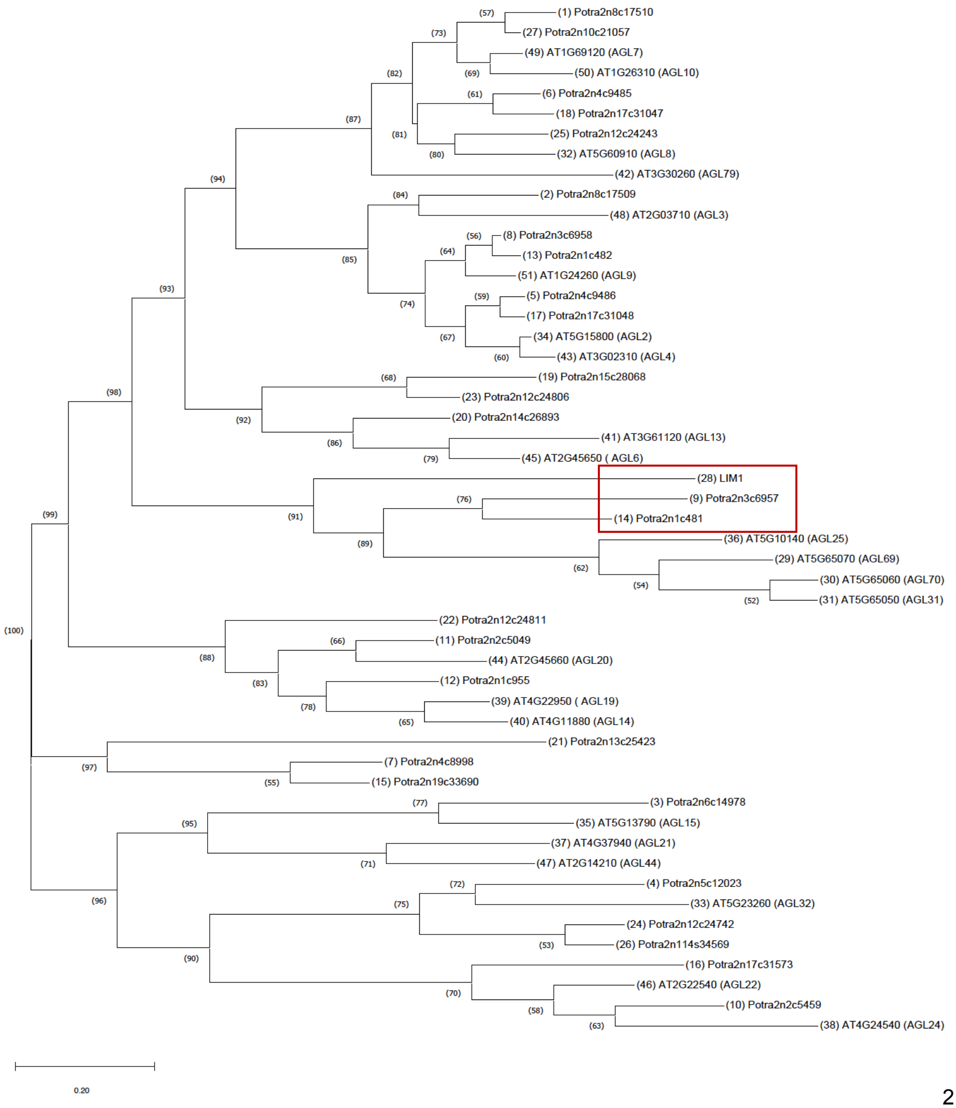

Appendix Figure S1. Domain structure and phylogenetic analysis of LIM1.

(A) The amino acid sequence and domain structure of LIM1. The N-terminal MADS-box domain is marked in teal and the K-box in grey colour. (B) Maximum likelihood Phylogeny tree (Bootstrap 1000) of LIM1 proteins of *Arabidopsis thaliana* and *Populus tremula*. The tree was constructed using MEGAX software. Scale bar indicates the average number of substitutions per site.

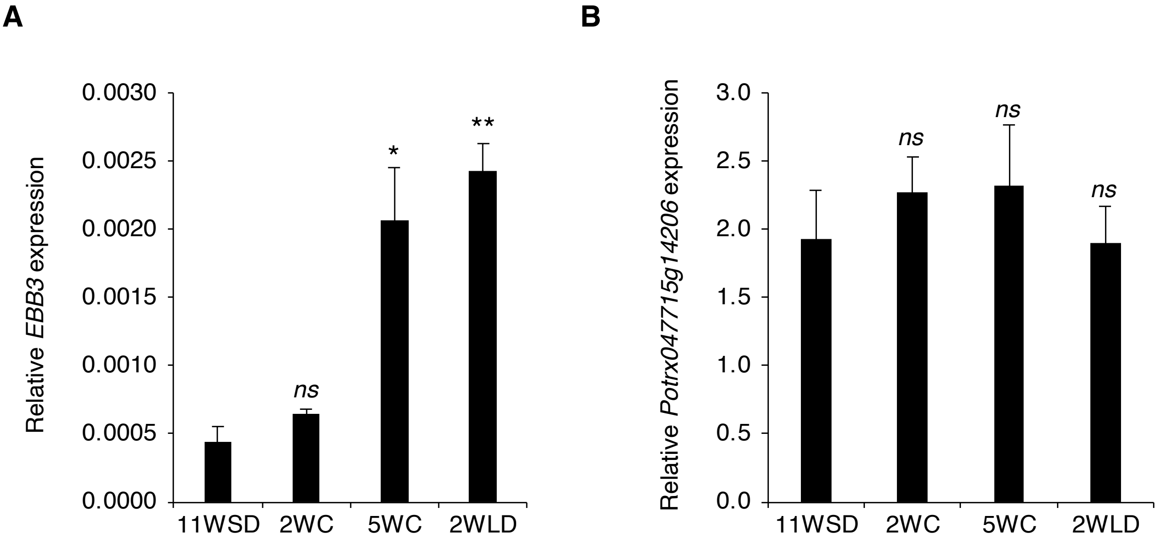

Appendix Figure S2. Expression pattern of positive and negative control genes.

The expression pattern of *EBB3* (A) and *Potrx047715g14206* (B) in wild-type buds, used as a positive and negative control, respectively. Expression values shown are normalized to the reference gene UBQ and are averages of three biological replicates. Error bars indicate standard error mean (± SEM). Asterisks indicate significant difference (**P*<0.05 and ***P*<0.01) with respect to 11WSD and *ns* indicates not significant. *P* values = 0.298 (11WSD vs 5WC) and 0.0021 (11WSD vs 2WLD). Statistical analysis was done using unpaired t-test.

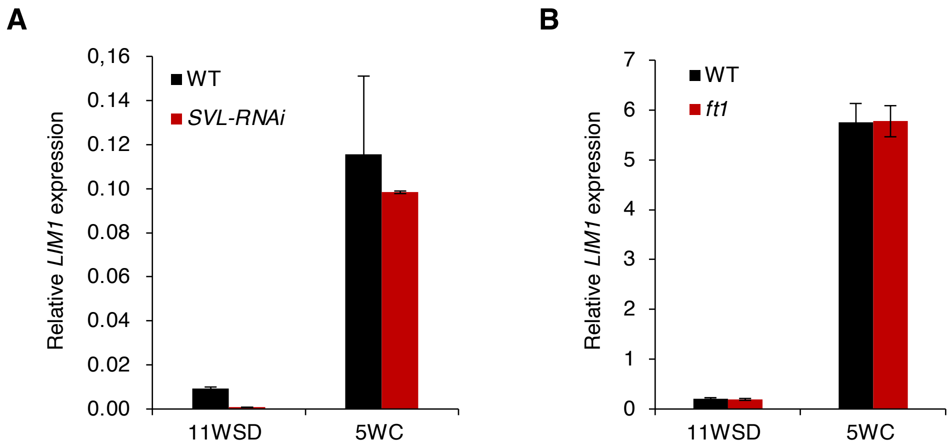

Appendix Figure S3. Activation of *LIM1* by LT is independent of *SVL* and *FT1.*

(A) *LIM1* expression in wild-type (T89) and *SVL-RNAi* line at different time points. (B) *LIM1* expression in WT and *ft1* line after 11 weeks of short-day (11WSD) and 5 weeks of cold temperature treatment (5WC). Expression values shown are normalized to the reference gene UBQ and are averages of three biological replicates (± SEM).

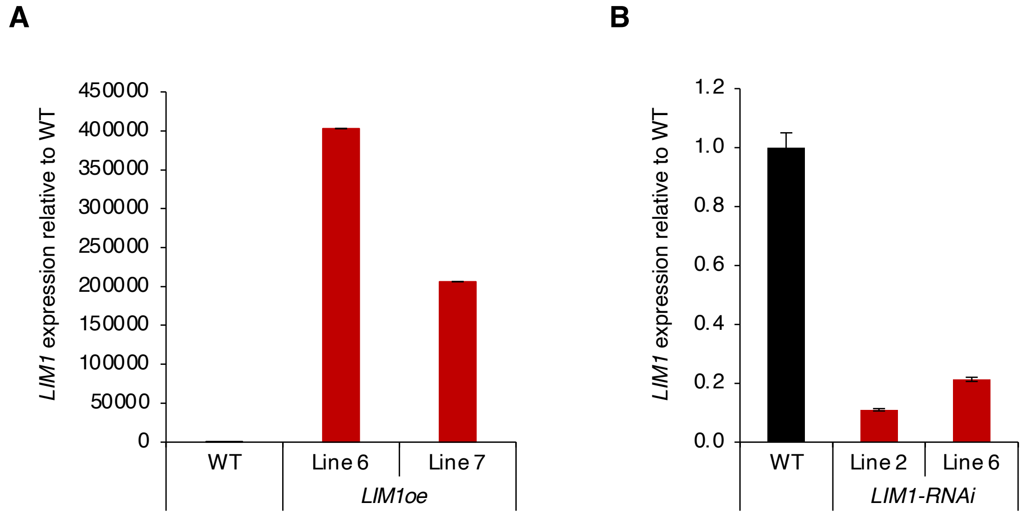

Appendix Figure S4. Generation of LIM1oe and LIM1-RNAi transgenic plants.

(A and B) *LIM1* expression in *LIM1* overexpressing (A) and RNAi (B) lines. See Figure 2. Expression values shown are normalized to the reference gene UBQ and are averages of three biological replicates (± SEM).

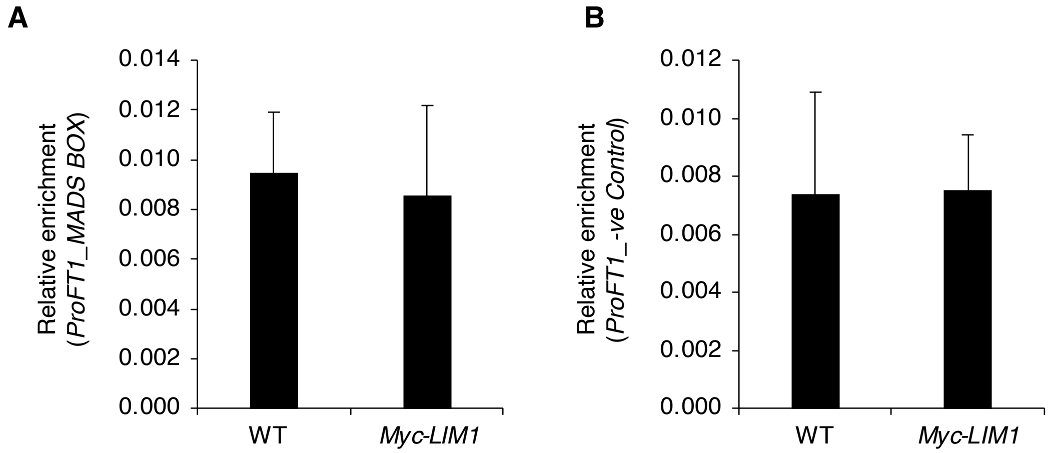

Appendix Figure S5. LIM1 binding on MADS-BOX motif in *FT1* promoter.

Enrichment of the DNA fragment with MADS-BOX motif (A) and without MADS-BOX motif (B) in the *FT1* promoter, quantified by chromatin immunoprecipitation (ChIP)-quantitative polymerase chain reaction (q-PCR). Expression values are represented as the percentage of input (% of input) DNA. Bars show average values from three independent biological replicates ± SEM.

A

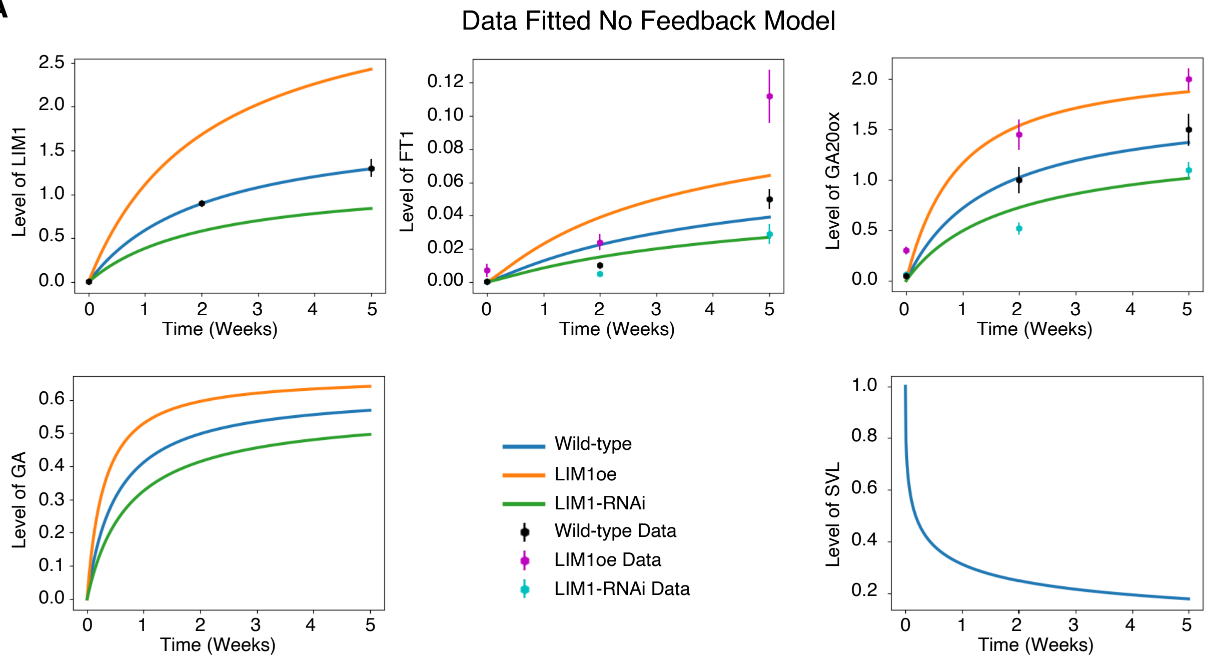
Data Fitted No Feedback Model
Level of LIM1
Level of FT1
Level of GA20ox
Level of GA
Level of SVL
Time (Weeks)
Wild-type
LIM1oe
LIM1-RNAi
Wild-type Data
LIM1oe Data
LIM1-RNAi Data

B

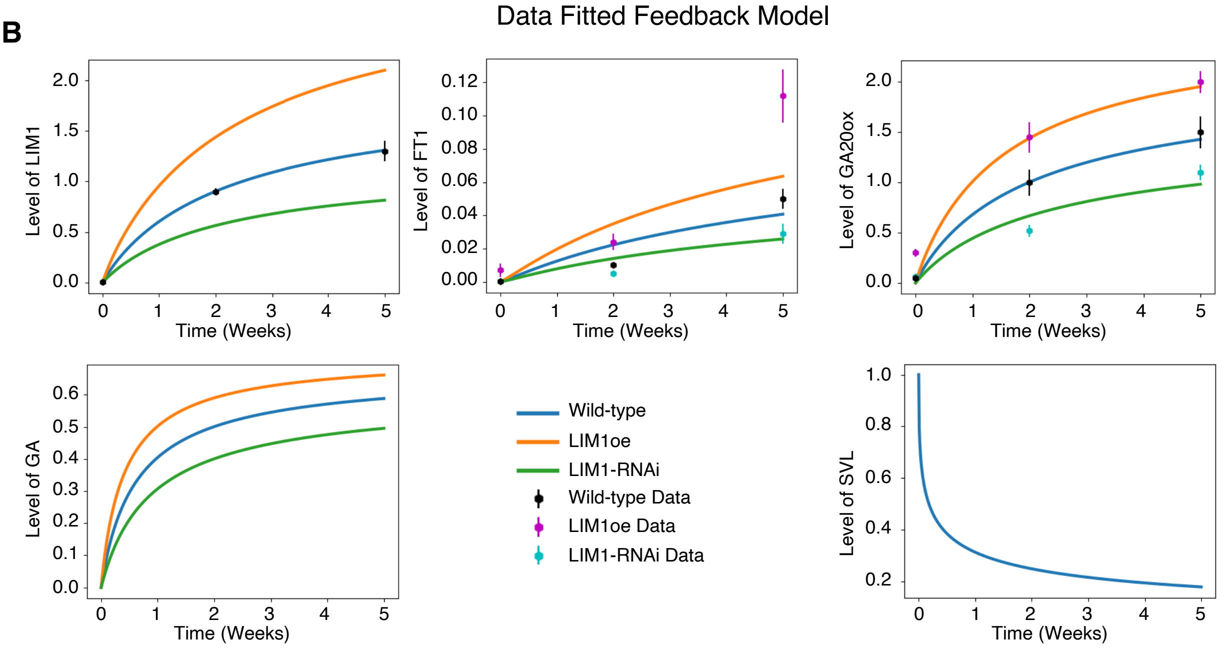
Data Fitted Feedback Model
Level of LIM1
Level of FT1
Level of GA20ox
Level of GA
Level of SVL
Time (Weeks)
Wild-type
LIM1oe
LIM1-RNAi
Wild-type Data
LIM1oe Data
LIM1-RNAi Data

Appendix Figure S6. Model predictions for wild-type, *LIM1* overexpression, and *LIM1-RNAi* plants.

The panels show the predicted dynamics of LIM1, FT1, GA20ox, GA and SVL after the onset of LT, using model parameters given in Table S1. Lines show the predictions for wild type (blue), *LIM1* over-expression (orange) and *LIM1-RNAi* (green). Dots and error bars show the *LIM1*, *FT1* and *GA20ox* experimental data for wild-type (black), LIM1-overexpression (purple) and LIM1-RNAi (turquoise). (A) Predictions of the model without feedback. (B) Predictions of the model with feedback (GA inhibiting *LIM1* expression).

Appendix Model Information

In the mathematical model, we work with the following system of ordinary differential equations (ODEs):

$$\frac{\mathrm{d}}{\mathrm{d}t}[\mathrm{LIM1}] = \alpha_{\mathrm{LIM1}} - \beta_{\mathrm{LIM1}}[\mathrm{LIM1}] \tag{1}$$

$$\frac{\mathrm{d}}{\mathrm{d}t}[\mathrm{FT1}] = \frac{\alpha_{\mathrm{FT1}} k_1^{\mathrm{FT1}}[\mathrm{LIM1}]}{1 + k_1^{\mathrm{FT1}}[\mathrm{LIM1}] + k_2^{\mathrm{FT1}}[\mathrm{SVL}] + k_3^{\mathrm{FT1}}[\mathrm{LIM1}][\mathrm{SVL}]} - \beta_{\mathrm{FT1}}[\mathrm{FT1}] \tag{2}$$

$$\frac{\mathrm{d}}{\mathrm{d}t}[\mathrm{GA20ox}] = \frac{\alpha_{\mathrm{GA20ox}}(k_1^{\mathrm{GA20ox}}[\mathrm{FT1}] + k_2^{\mathrm{GA20ox}}[\mathrm{LIM1}] + k_3^{\mathrm{GA20ox}}[\mathrm{FT1}][\mathrm{LIM1}])}{1 + k_1^{\mathrm{GA20ox}}[\mathrm{FT1}] + k_2^{\mathrm{GA20ox}}[\mathrm{LIM1}] + k_3^{\mathrm{GA20ox}}[\mathrm{FT1}][\mathrm{LIM1}]} - \beta_{\mathrm{GA20ox}}[\mathrm{GA20ox}] \tag{3}$$

$$\frac{\mathrm{d}}{\mathrm{d}t}[\mathrm{GA}] = \frac{\alpha_{\mathrm{GA}}[\mathrm{GA20ox}]}{k_1^{\mathrm{GA}} + [\mathrm{GA20ox}]} - \beta_{\mathrm{GA}}[\mathrm{GA}] \tag{4}$$

$$\frac{\mathrm{d}}{\mathrm{d}t}[\mathrm{SVL}] = \alpha_{\mathrm{SVL}} - \beta_{\mathrm{SVL}}[\mathrm{SVL}] \tag{5}$$

Here, each equation models the rate of change of each variable. In equations (1-5), the final terms represent the degradation of each variable, where each β determines the degradation rate, while the first terms represent production, with the α's denoting the maximum production rate of each variable. In equations (2-4), we use the standard Hill terms to represent the regulation of the production rates [1, 2]. The first term of (2) models both FT1 promotion by LIM1 and FT1 inhibition by SVL, with the $k_3^{\mathrm{FT1}}[\mathrm{LIM1}][\mathrm{SVL}]$ part representing the combined effect of the inhibition and promotion of FT1. GA20ox is promoted by both FT1 and LIM1, this is modelled in the first term of (3), with the $k_3^{\mathrm{GA20ox}}[\mathrm{FT1}][\mathrm{LIM1}]$ part representing of the combined effect of the promotion of GA20ox by FT1 and LIM1. GA synthesis is mediated by GA20ox, this is represented in the production term in (4).

Given that signalling networks equilibrate over the timescale of hours, we expect the network of interactions to be in equilibrium over the weeks-long timescale of the experimental data. As these data show how LIM1 and SVL levels vary over the timescale of weeks, we define the maximum production rates of LIM1 and SVL (in equations (6) and (7) respectively), in terms of the time variable T which spans the timescale of weeks, and is distinct from t, which varies over the timescale of hours.

$$\alpha_{\mathrm{LIM1}}(T) = \frac{\alpha_{\mathrm{LIM1}}^{max} T}{\gamma_{\mathrm{LIM1}} + T} \tag{6}$$

$$\alpha_{\mathrm{SVL}}(T) = \frac{\alpha_{\mathrm{SVL}}^{max} \gamma_{SVL}^n}{\gamma_{SVL}^n + T^n} \tag{7}$$

To create simulations of the model, we calculated the steady states of the core interaction network (1-5) as α_{LIM1} and α_{SVL} vary over the long timescale, according to (6,7). The model was coded and simulated in python and the steady states of equations (1-5) were calculated using the `fsolve` function from [3].

As described in the main text, having considered the network configuation represented by equations (1-5), we then extended the network to include GA inhibiting LIM1. This extended model is given by equations (2-5) but with equation (1) replaced with

$$\frac{\mathrm{d}}{\mathrm{d}t}[\mathrm{LIM1}] = \frac{\alpha_{\mathrm{LIM1}} k_1^{\mathrm{LIM1}}}{k_1^{\mathrm{LIM1}} + [\mathrm{GA}]} - \beta_{\mathrm{LIM1}}[\mathrm{LIM1}] \tag{8}$$

Here, LIM1 production is modelled via a Hill function, with GA present in the denominator to represent the inhibition of LIM1 by GA.

Both models were fitted to the experimental data, for the wild-type, LIM1oe and LIM1-RNAi plants, totalling 21 data points. To simulate LIM1oe and LIM1-RNAi, we multiplied α_{LIM1} by a parameter in each case, corresponding to the increased LIM1 production rate in LIM1oe, and the reduced LIM1 production rate in LIM1-RNAi. The values of these two parameters were estimated to ensure agreement between the model predictions and data as part of the parameter estimation processes. To estimate the parameter values, we used the least squares method and the Metropolis Hastings algorithm to identify parameter sets for which the model predictions best agree with the experimental data. The final parameter estimates were found using the `leastsq` algorithm from [4] (see Table S1). Whilst this produced a parameter set with reasonable agreement between the predictions and data, some of the parameter estimates it provided were negative and thus unphysical, and needed to be adjusted by hand (albeit with little effect on the agreement between predictions and data). The estimated parameters revealed that fitting the model with no GA feedback required higher degradation rates and lower production rates when compared to the model with feedback.

Appendix Table S1: Description of Parameters used in this Model, where the last two columns are the values used to data fit the model without feedback and with feedback, respectively

Parameter	Definition	No Feedback	Feedback
$\alpha_{\text{LIM1}}^{max}$	Maximum production rate of LIM1 on the long timescale	1.32	1.32
γ_{LIM1}	Half Maximum Threshold for α_{LIM1}	2.1	2.5
k_1^{LIM1}	Magnitude of LIM1 inhibition by GA	N/A	2
β_{LIM1}	Degradation rate of LIM1	0.72	0.52
α_{FT1}	Production rate of FT1	0.35	1.3
k_1^{FT1}	Magnitude of FT1 regulation by LIM1	0.7	0.12
k_2^{FT1}	Magnitude of FT1 inhibition by SVL	20	15
k_3^{FT1}	Magnitude of the combined regulation/inhibition of FT1 inhibition by LIM1 and SVL	0.01	0.1
β_{FT1}	Degradation rate of FT1	1.48	1.3
α_{GA20ox}	Maximum production rate of GA20ox	1.5	1.3
k_1^{GA20ox}	Magnitude of GA20ox regulation by FT1	0.1	3.2
k_2^{GA20ox}	Magnitude of GA20ox regulation by LIM1	0.54	0.29
k_3^{GA20ox}	Magnitude of GA20ox regulation by both FT1 and LIM1	8.49	3.3
β_{GA20ox}	Degradation rate of GA20ox	0.58	0.37
α_{GA}	Maximum production rate of GA	1	1
k_1^{GA}	Magnitude of GA regulation by GA20ox	1	1
β_{GA}	Degradation rate of GA	1	1
$\alpha_{\text{SVL}}^{max}$	Maximum production rate of SVL on the long timescale	1	1
γ_{SVL}	Half Maximum Threshold for α_{SVL}	0.18	0.18
n	Hill exponent of α_{SVL}	0.46	0.46
β_{SVL}	Degradation rate of SVL	1	1

Appendix Table S2. Primers used in the study

Experiment	Oligos	Sequence (5`- 3`)
Real time PCR	*PttUBQ-F*	GTTGATTTTTGCTGGGAAGCG
	PttUBQ-R	GATCTTGGCCTTCACGTTGT
	PttLIM1-F	CTCCAAATGGTCCAAAGGTAC
	PttLIM1-R	GCTTCTGTCTTTCTTCCTCTGG
	PttGA20ox1-F	TTCCACAACGAGAGCGGTCTTG
	PttGA20ox1-R	TTTGAGAGGCAGGGAAGGGAGAG
	GA20ox1_ChIP-F	GCACTCTCTTGATACAAGGCA
	GA20ox1_ChIP-R	TCCTCAATGAGAAAACTTGGTGA
	PttFT1-F	GCAAGCTTTGGCCATGAAAC
	PttFT1-R	GGATATCTTCCTGTTATCGC
Cloning	*LIM1-FL-FP*	CACCATGGTGAGAGGGAAGGTGCAGTTAC
	LIM1-FL-RP	CTAAAAGTGGAACAAGTCCAGTGGAG
	LIM1-RNAi-FP	CACCATGGTGAGAGGGAAGGTGCAGTTAC
	LIM1-RP-RNAi	GTATTCTGCGTGATGATTCTGCTTG
	FT1-CRISPR-SgRNA3F	ATATATGGTCTCGATTGTGCGAGCTCAAACCCTCT CGTTTTAGAGCTAGAAATAGC
	FT1-CRISPR-SgRNA3R	ATTATTGGTCTCTAAACGCCAATATCAACCCTCGG C CAATCTCTTAGTCGACTCTAC
Screening of transgenic lines	*PttLIM1-F1*	CTCCAAATGGTCCAAAGGTAC
	PttLIM1-R1	GCTTCTGTCTTTCTTCCTCTGG
	FT1-CRISPR-F	CACCATGTCAAGGGATAGAGATCCTC
	FT1-CRISPR-R	GTGCATCAGGGTCCACCATAAC
Y1H constructs	*PttLIM1-F*	CTCCAAATGGTCCAAAGGTAC
	PttLIM1-R	GCTTCTGTCTTTCTTCCTCTGG
	p4p1r ProGA20ox-F	GGGGACAACTTTGTATAGAAAAGTTGTAAAGATC AAACACTATTGCAAACAAAAAAGGAG
	p4p1r ProGA20ox-R	GGGGACTGCTTTTTTGTACAAACTTGTTGCATTAC GAATTTGTGACTGGTGTG

References

Bintu, L., Buchler, N.E., Garcia, H.G., Gerland, U., Hwa, T., Kondev, J. and Phillips, R., 2005. Transcriptional regulation by the numbers: models. Current opinion in genetics & development, 15(2), pp.116-124.

Harris, C.R., Millman, K.J., van der Walt, S.J. et al., 2020 Array programming with NumPy. Nature 585, pp. 357–362

Jones, E., and Oliphant, T., Peterson P. et al., SciPy: Open source scientific tools for Python, 2001â€“, http://www.scipy.org/

Klipp, E., Liebermeister, W., Wierling, C. and Kowald, A., 2016. Systems biology: a textbook. John Wiley & Sons.
