# Supplementary figures and images for: A regulatory module mediating temperature control of cell-cell communication facilitates tree bud dormancy release
Source: EMBO J. 2024 Oct 3;43(23):4. doi: 10.1038/s44318-024-00256-5 (PMC11612439; doi:10.1038/s44318-024-00256-5)

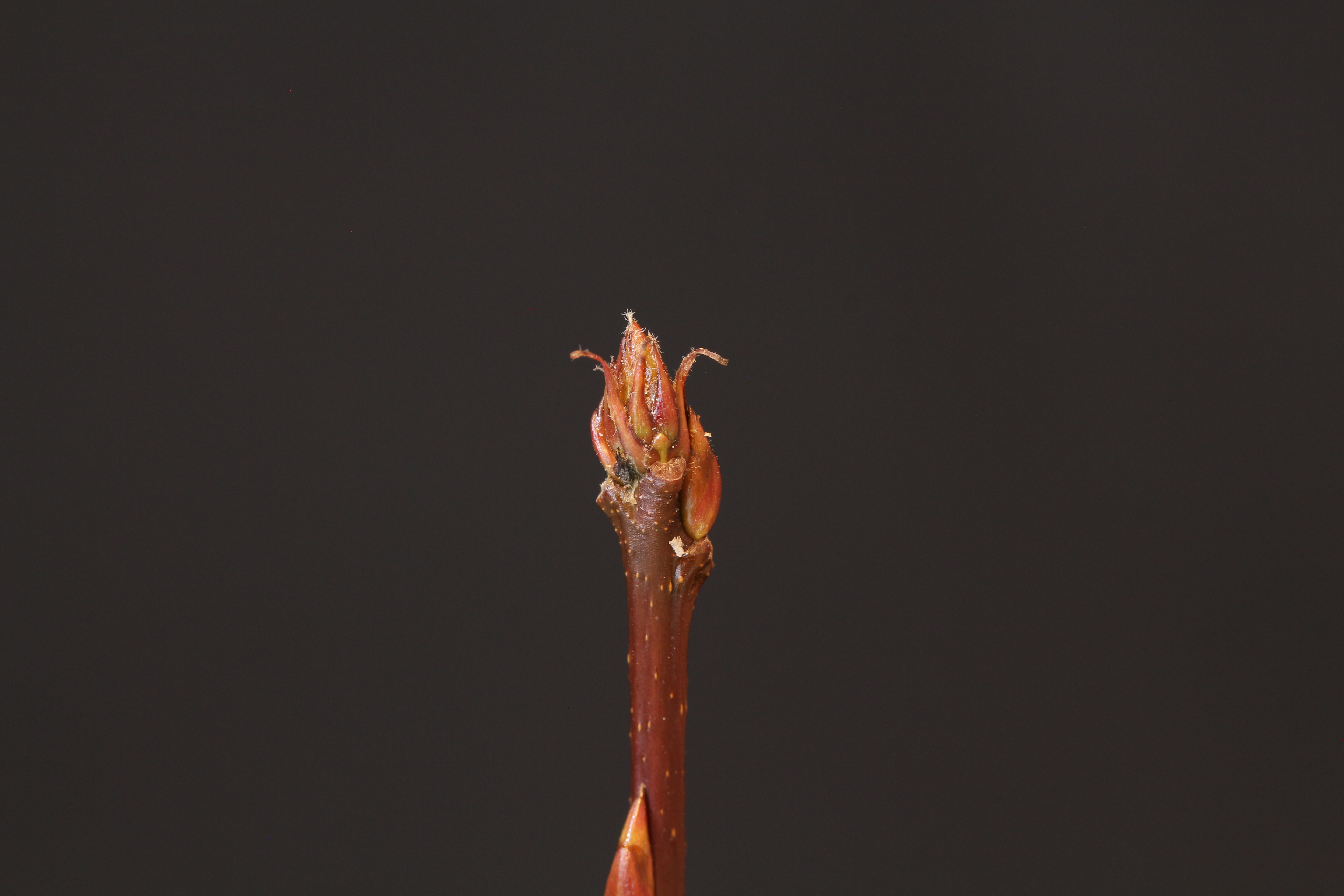

Supplement: Supplementary file 4 — Source data Fig. 2 [file 44318_2024_256_MOESM4_ESM.zip › SD Figure 2/1. Fig 2A-C/A/1. 2A-WT.JPG]

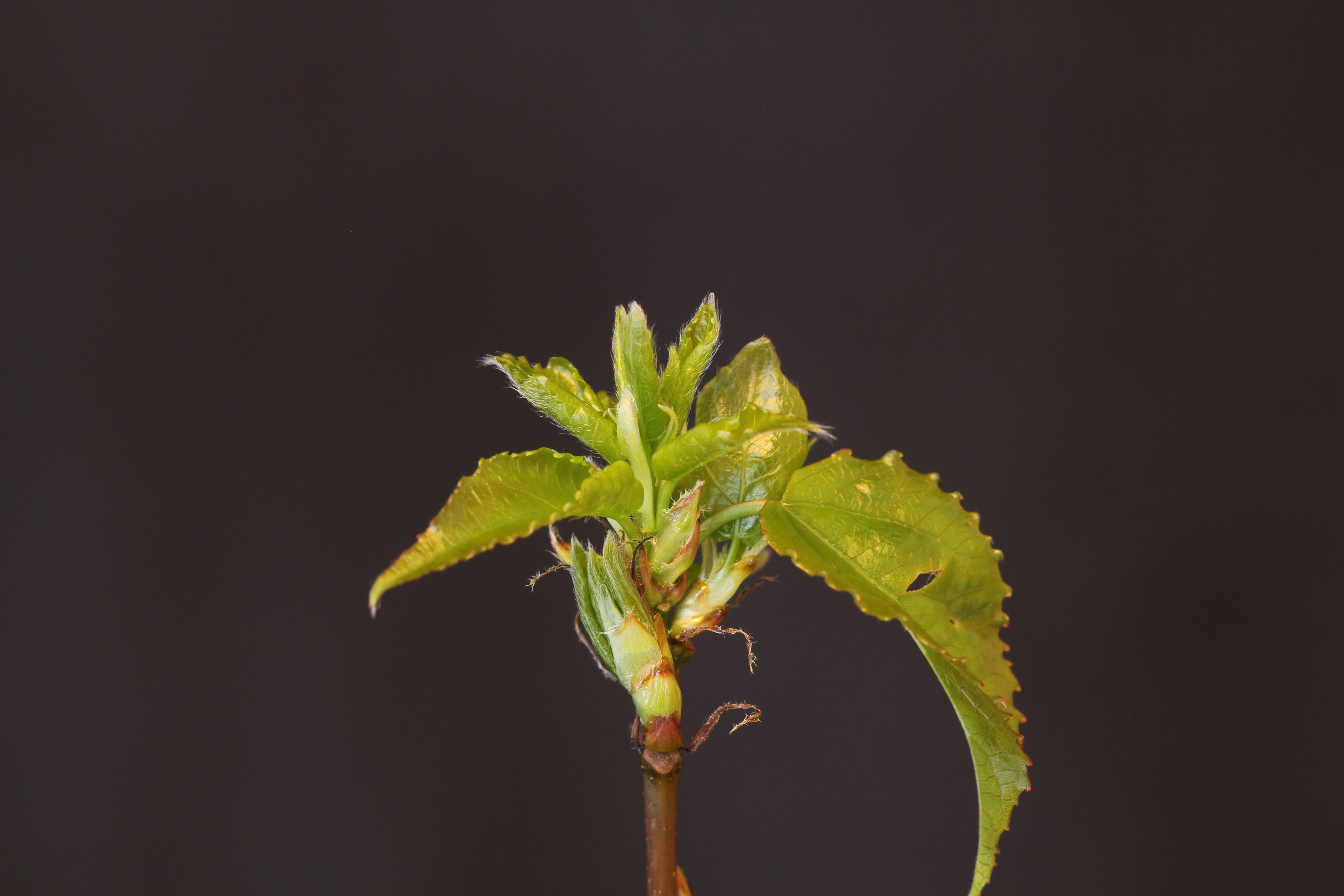

Supplement: Supplementary file 4 — Source data Fig. 2 [file 44318_2024_256_MOESM4_ESM.zip › SD Figure 2/1. Fig 2A-C/A/3. 2A-LIM1oe-L7.JPG]

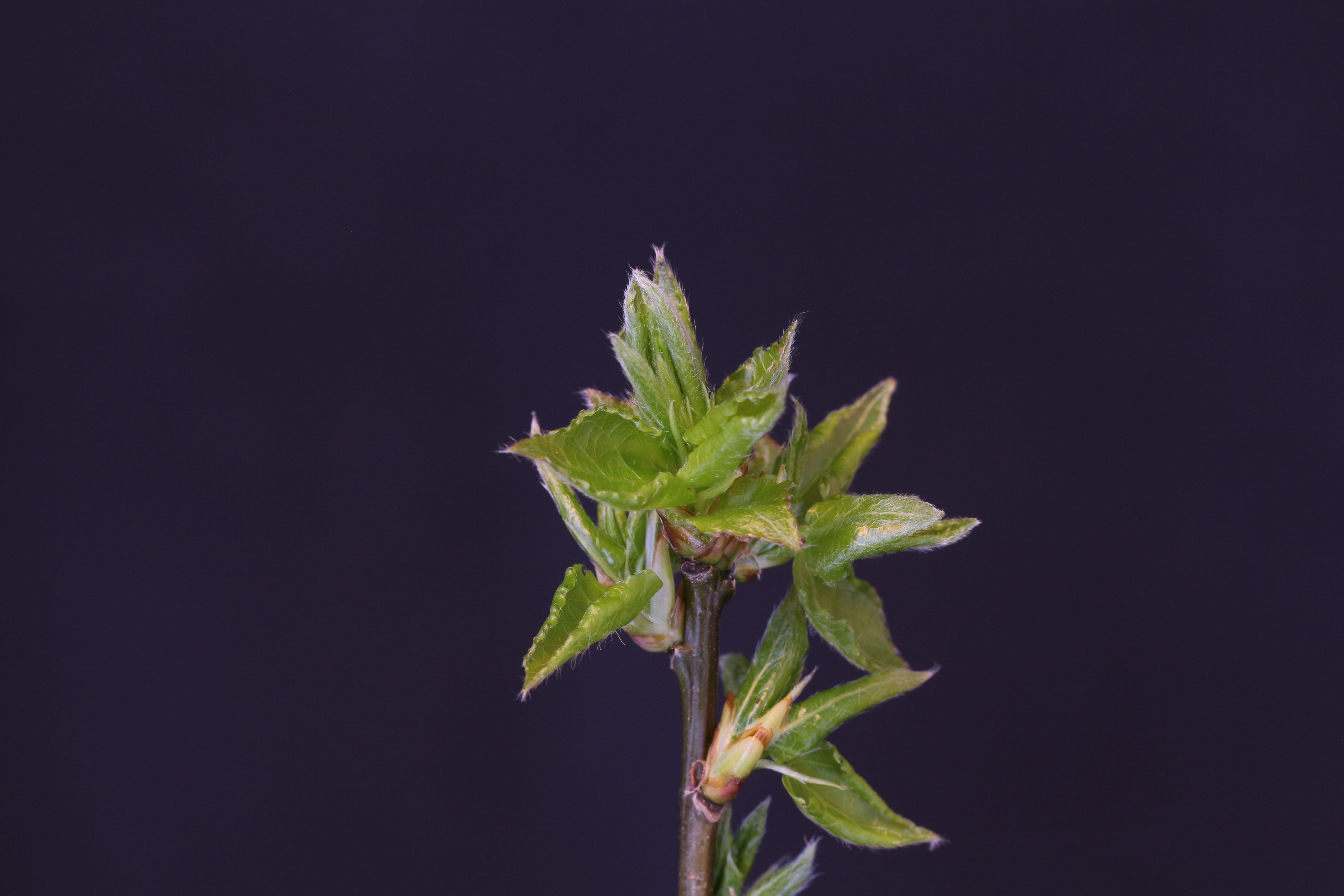

Supplement: Supplementary file 4 — Source data Fig. 2 [file 44318_2024_256_MOESM4_ESM.zip › SD Figure 2/1. Fig 2A-C/A/2. 2A-LIM1oe-L6.JPG]

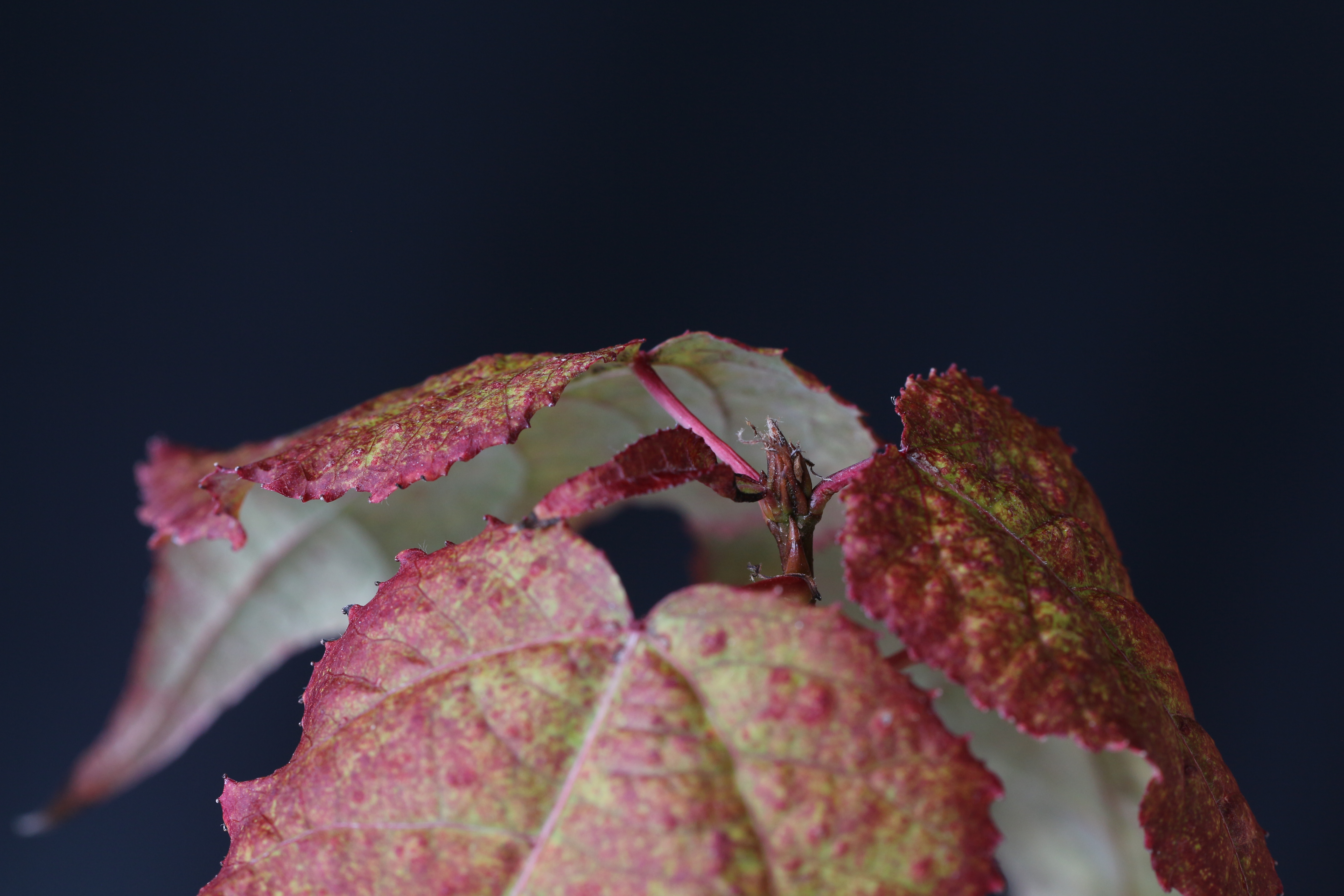

Supplement: Supplementary file 4 — Source data Fig. 2 [file 44318_2024_256_MOESM4_ESM.zip › SD Figure 2/1. Fig 2A-C/C/1. 2C-WT.JPG]

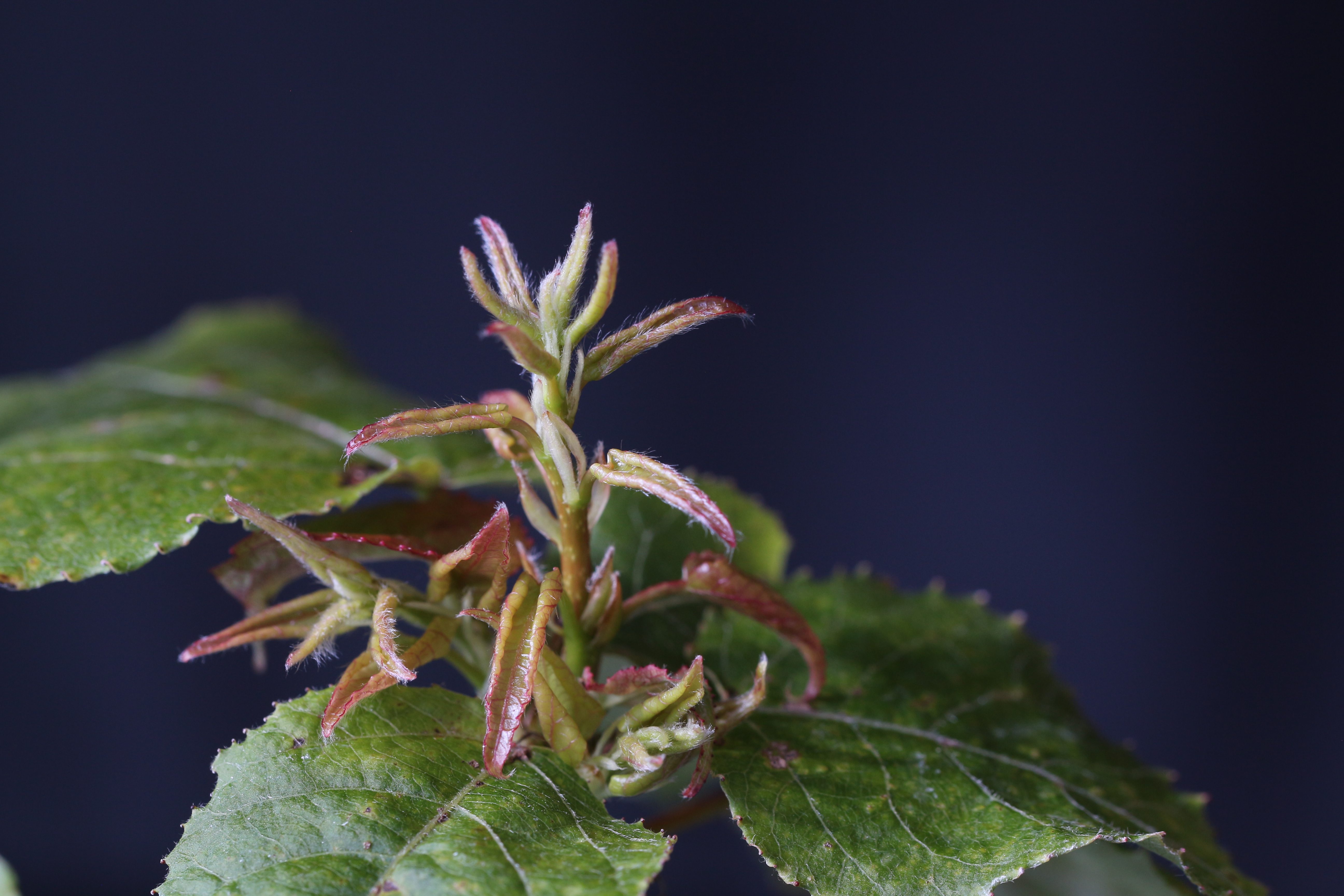

Supplement: Supplementary file 4 — Source data Fig. 2 [file 44318_2024_256_MOESM4_ESM.zip › SD Figure 2/1. Fig 2A-C/C/3. 2C-LIM1oe-L7.JPG]

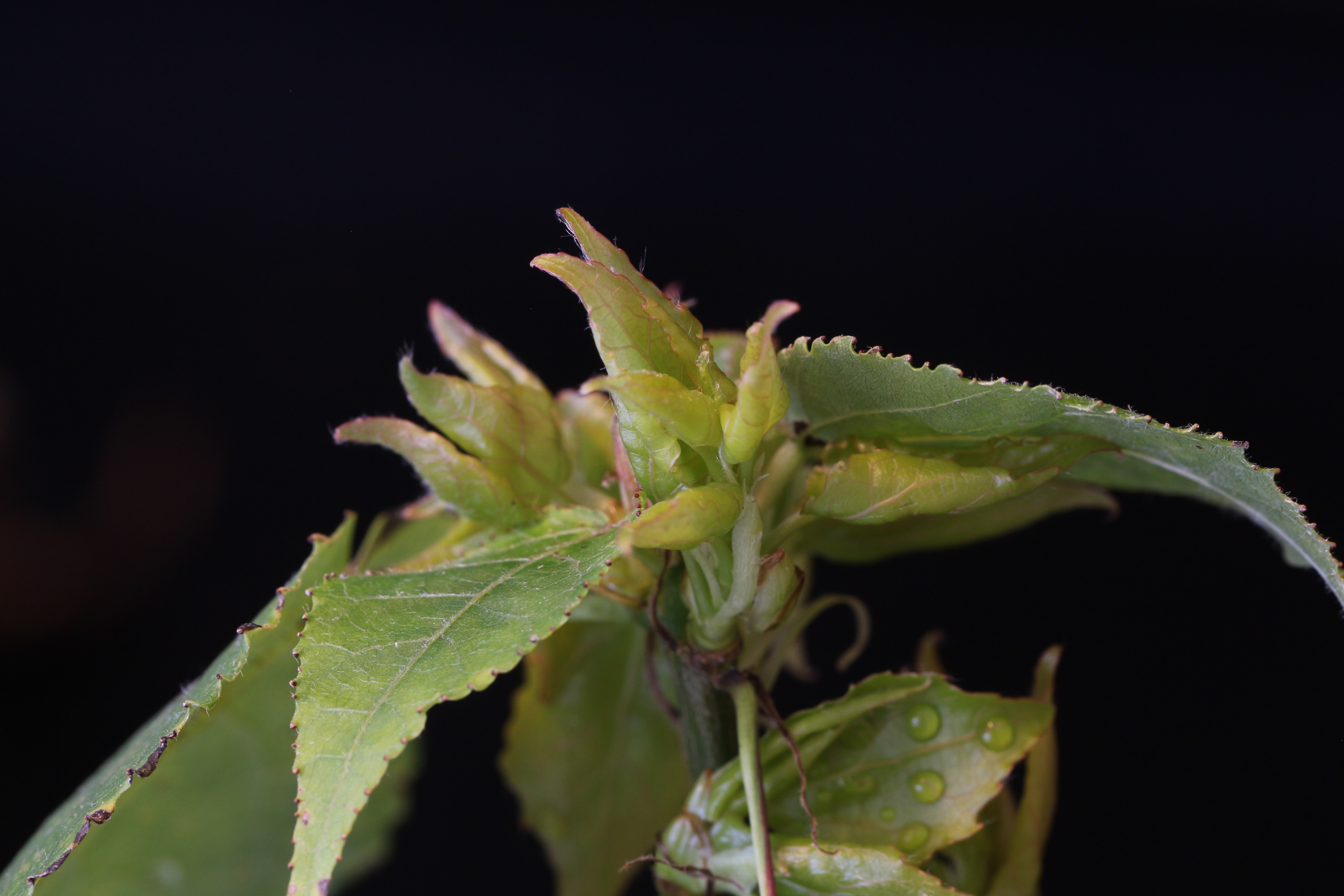

Supplement: Supplementary file 4 — Source data Fig. 2 [file 44318_2024_256_MOESM4_ESM.zip › SD Figure 2/1. Fig 2A-C/C/2 .2C-LIM1oe-L6.JPG]

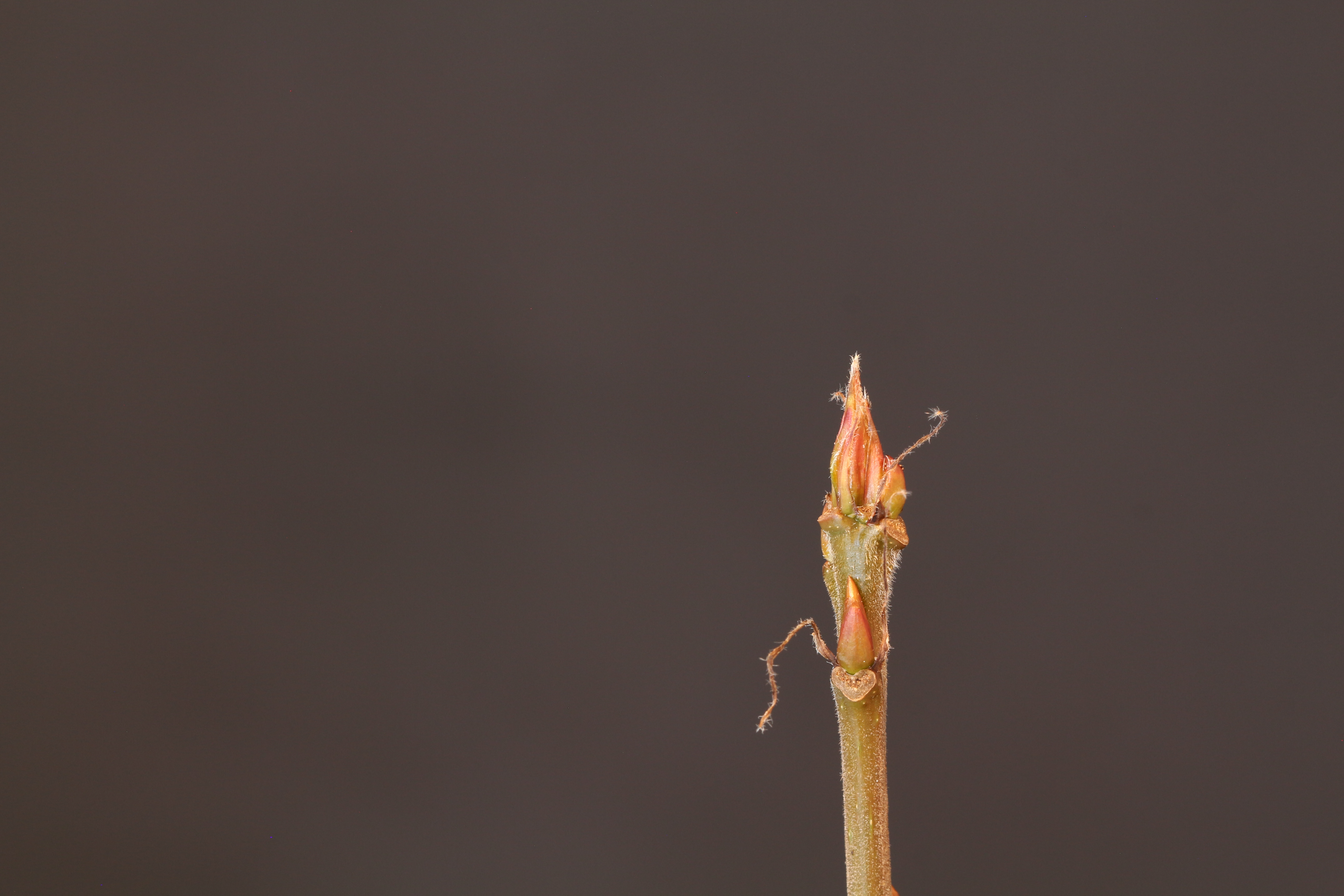

Supplement: Supplementary file 4 — Source data Fig. 2 [file 44318_2024_256_MOESM4_ESM.zip › SD Figure 2/1. Fig 2A-C/B/3. 2B-LIM1-RNAi-L6.JPG]

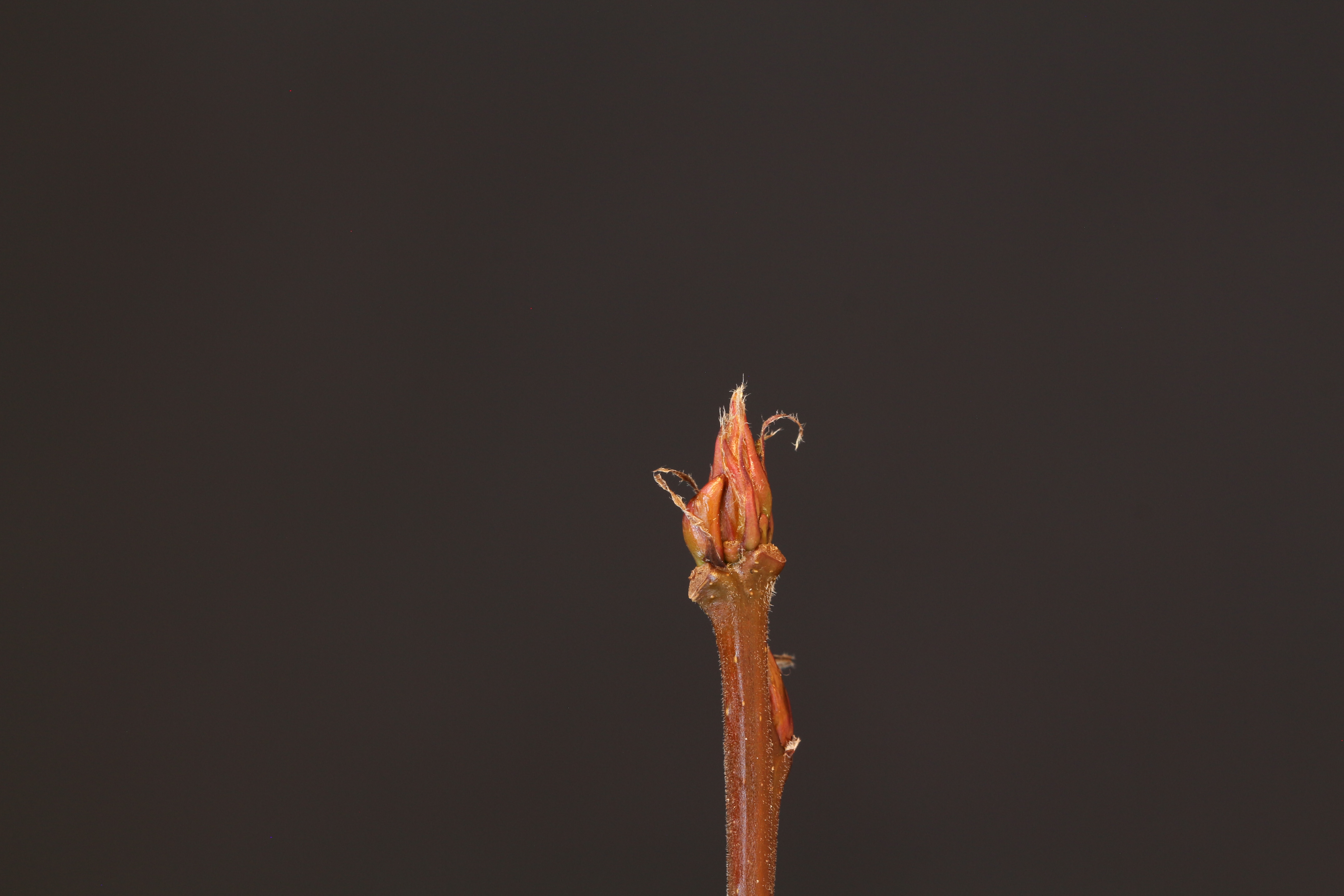

Supplement: Supplementary file 4 — Source data Fig. 2 [file 44318_2024_256_MOESM4_ESM.zip › SD Figure 2/1. Fig 2A-C/B/2. 2B-LIM1-RNAi-L2.JPG]

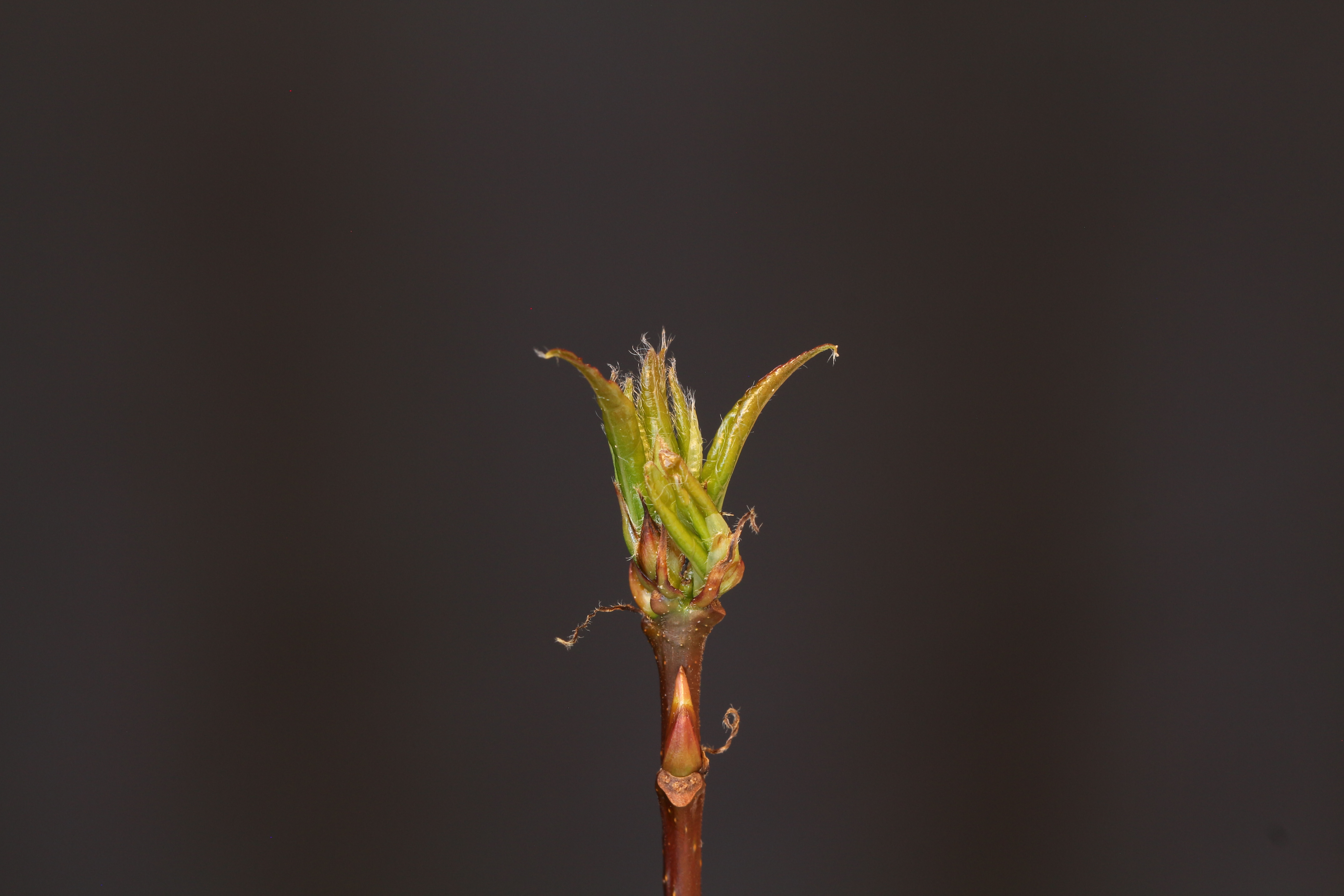

Supplement: Supplementary file 4 — Source data Fig. 2 [file 44318_2024_256_MOESM4_ESM.zip › SD Figure 2/1. Fig 2A-C/B/1. 2B-WT.JPG]

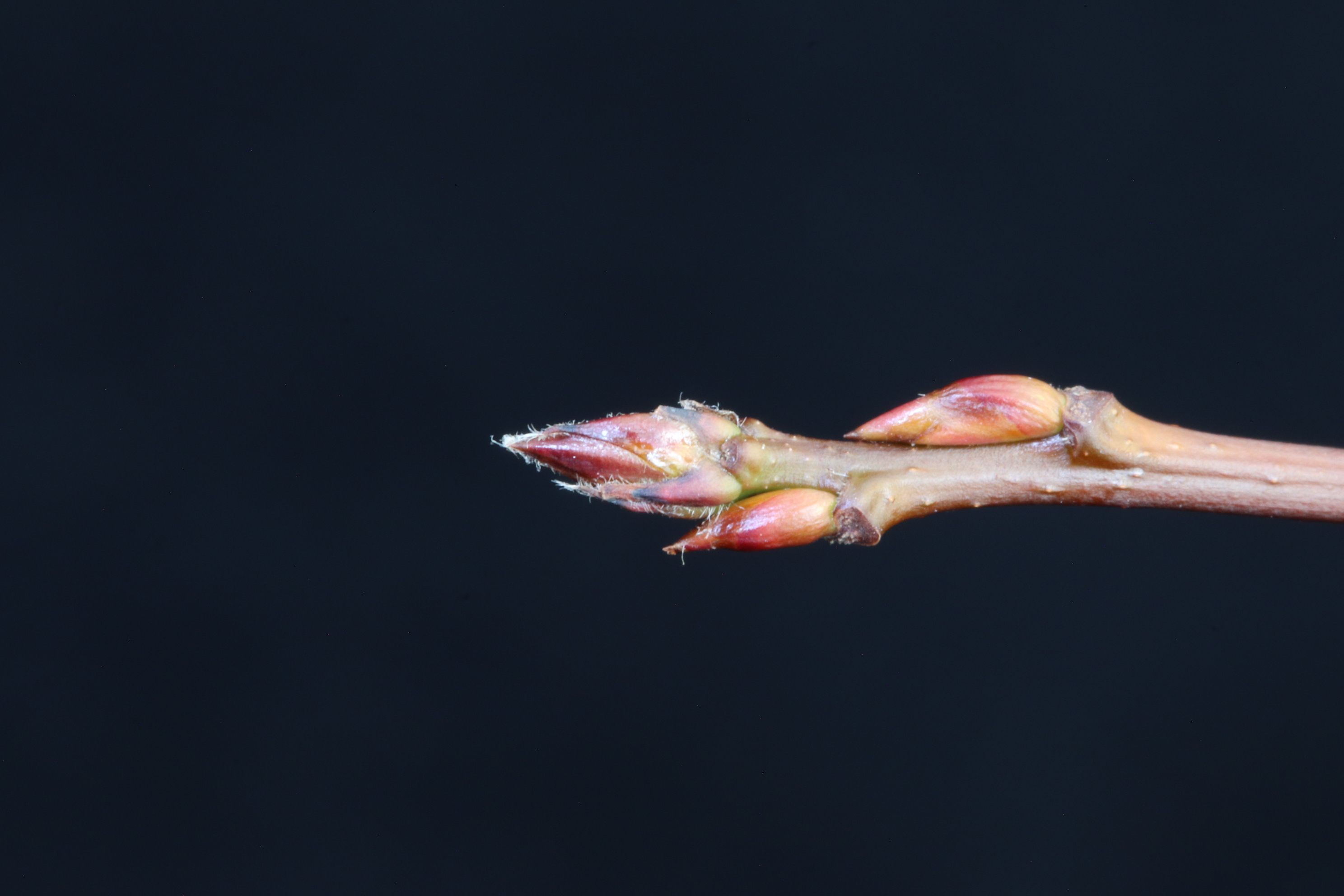

Supplement: Supplementary file 5 — Source data Fig. 3 [file 44318_2024_256_MOESM5_ESM.zip › SD Figure 3/Fig 3E/2. LIM1oe on FT1oe_1W.JPG]

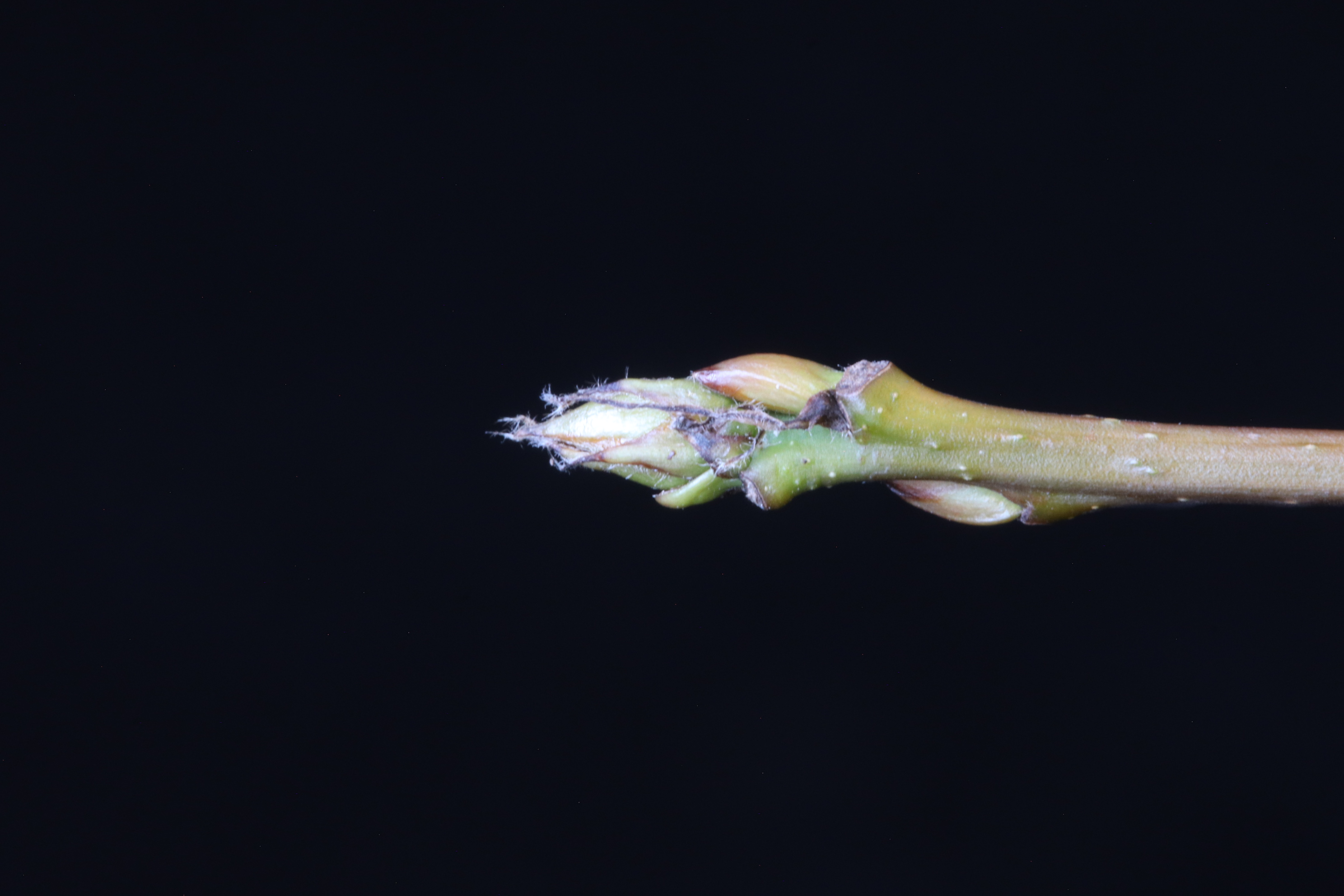

Supplement: Supplementary file 5 — Source data Fig. 3 [file 44318_2024_256_MOESM5_ESM.zip › SD Figure 3/Fig 3E/3. WT on FT1oe_5W.JPG]

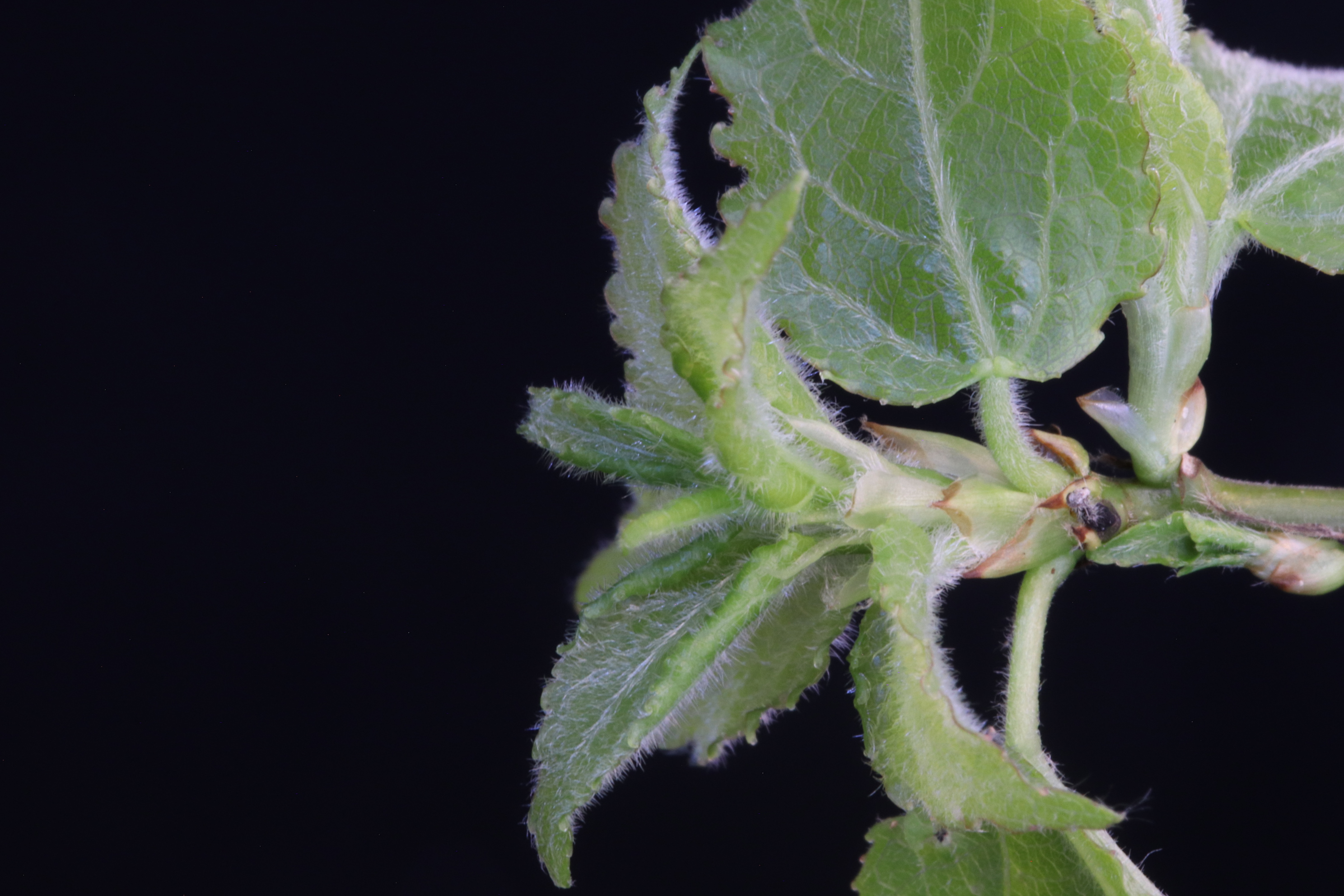

Supplement: Supplementary file 5 — Source data Fig. 3 [file 44318_2024_256_MOESM5_ESM.zip › SD Figure 3/Fig 3E/4. LIM1oe on FT1oe_5W.JPG]

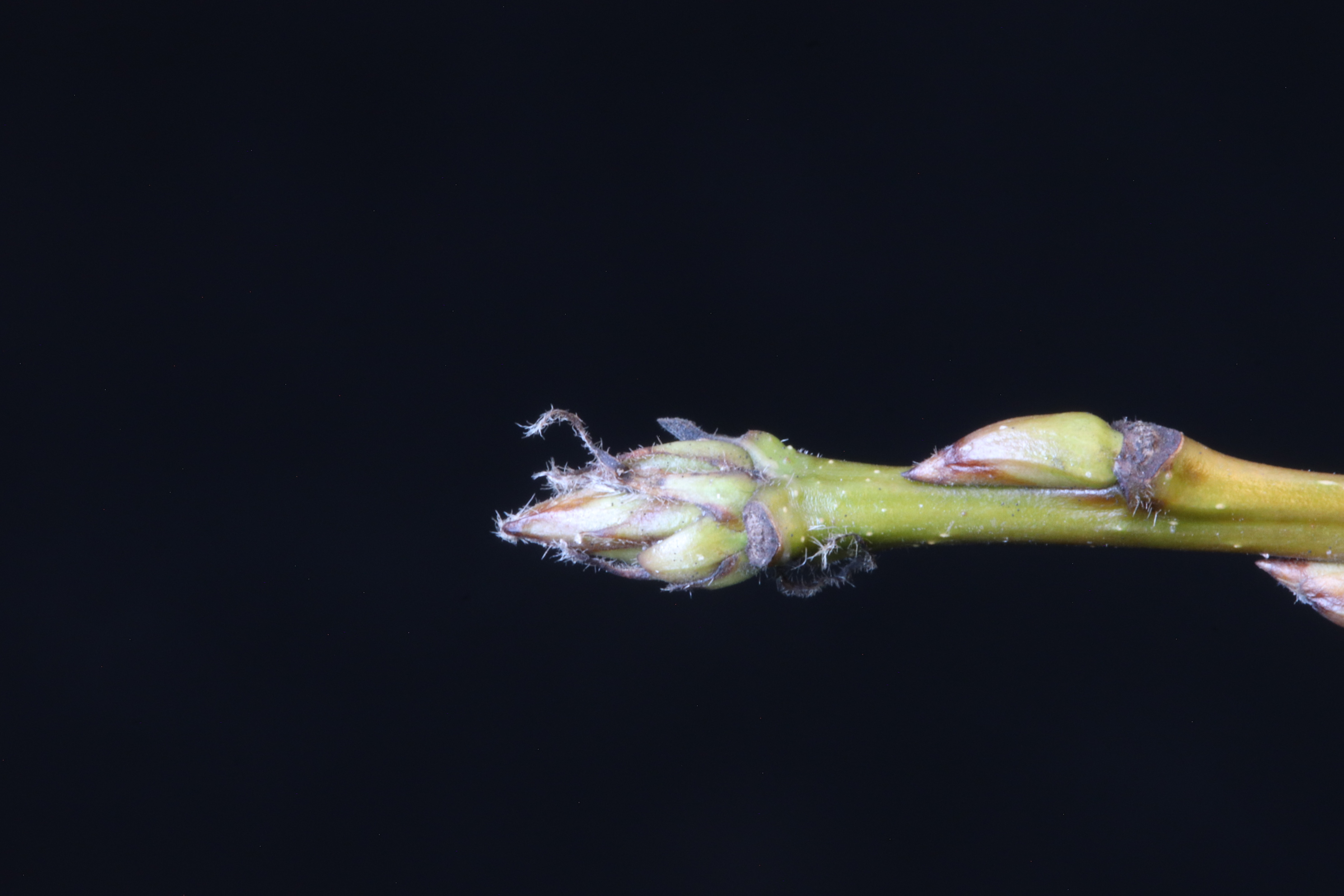

Supplement: Supplementary file 5 — Source data Fig. 3 [file 44318_2024_256_MOESM5_ESM.zip › SD Figure 3/Fig 3E/1. WT on FT1oe_1W.JPG]

## Slide 1
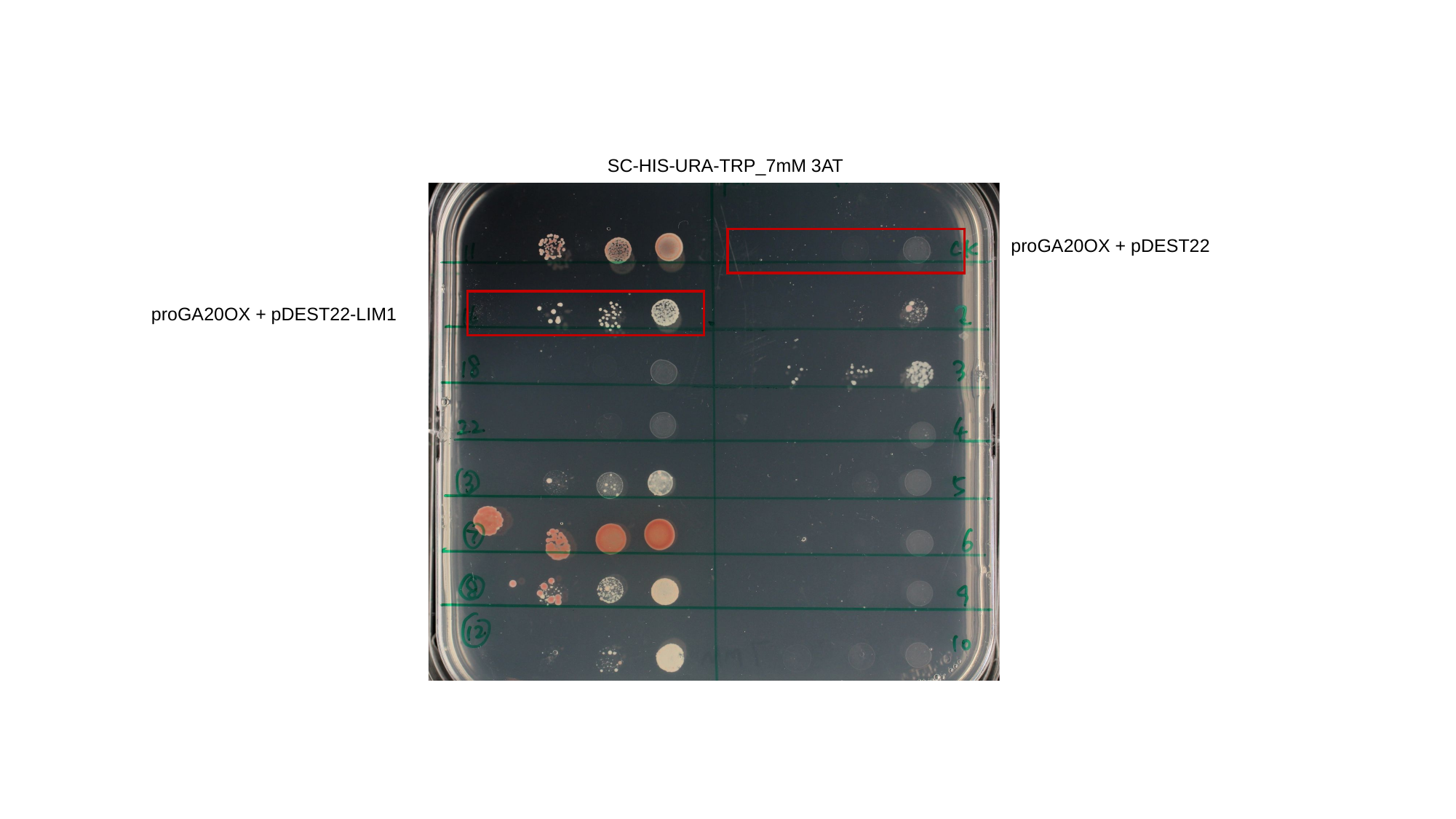

SC-HIS-URA-TRP_7mM 3AT
proGA20OX + pDEST22
proGA20OX + pDEST22-LIM1

Supplement: Supplementary file 6 — Source data Fig. 4 [file 44318_2024_256_MOESM6_ESM.zip › SD Figure 4/Fig 4C/Fig 4C.pptx]

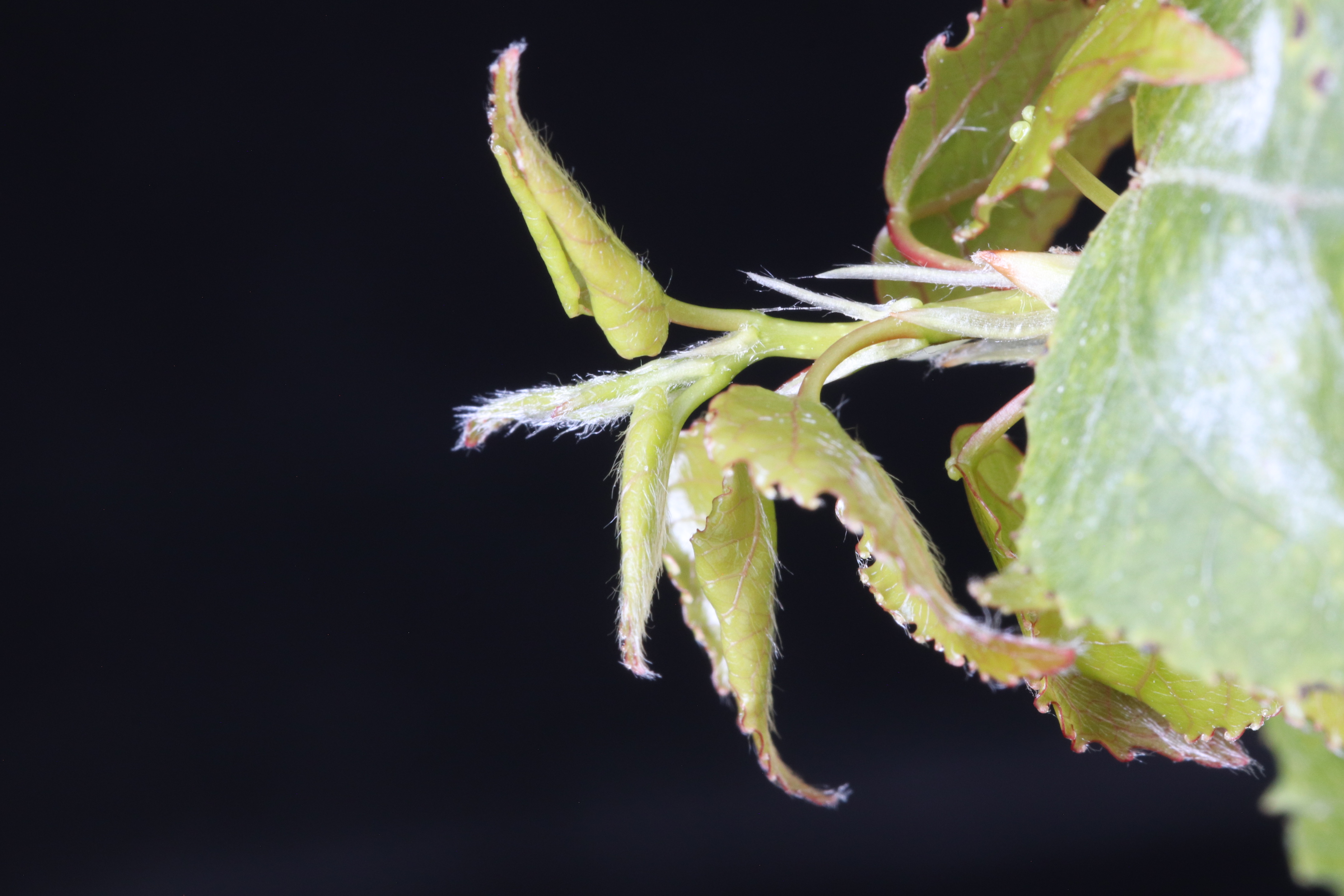

Supplement: Supplementary file 8 — Source data Fig. 6 [file 44318_2024_256_MOESM8_ESM.zip › SD Figure 6/Fig 6A-C/A/2. 6A-LIM1oe.JPG]

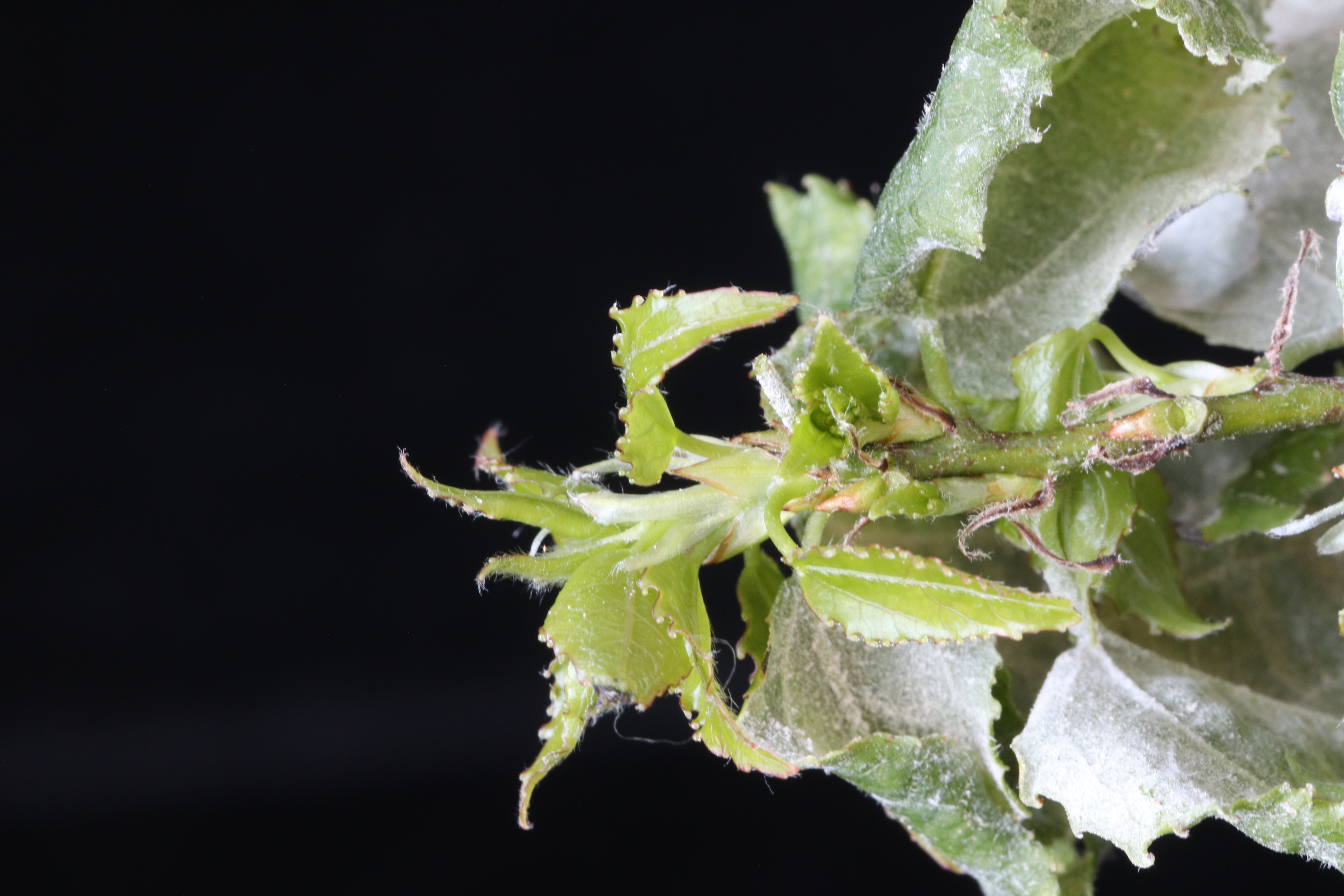

Supplement: Supplementary file 8 — Source data Fig. 6 [file 44318_2024_256_MOESM8_ESM.zip › SD Figure 6/Fig 6A-C/A/3. 6A-LIM1oe-ft1-3.JPG]

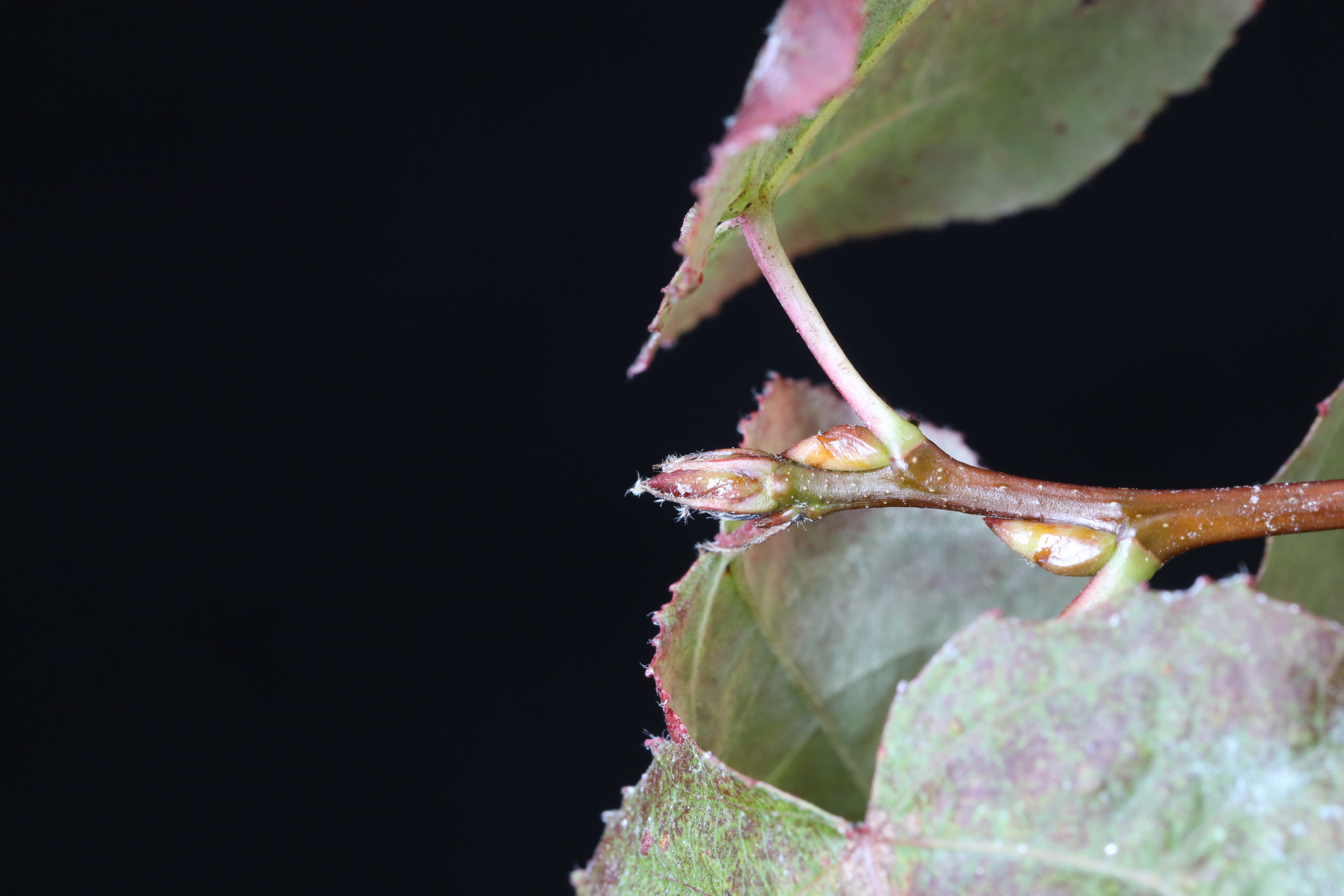

Supplement: Supplementary file 8 — Source data Fig. 6 [file 44318_2024_256_MOESM8_ESM.zip › SD Figure 6/Fig 6A-C/A/1. 6A-WT.JPG]

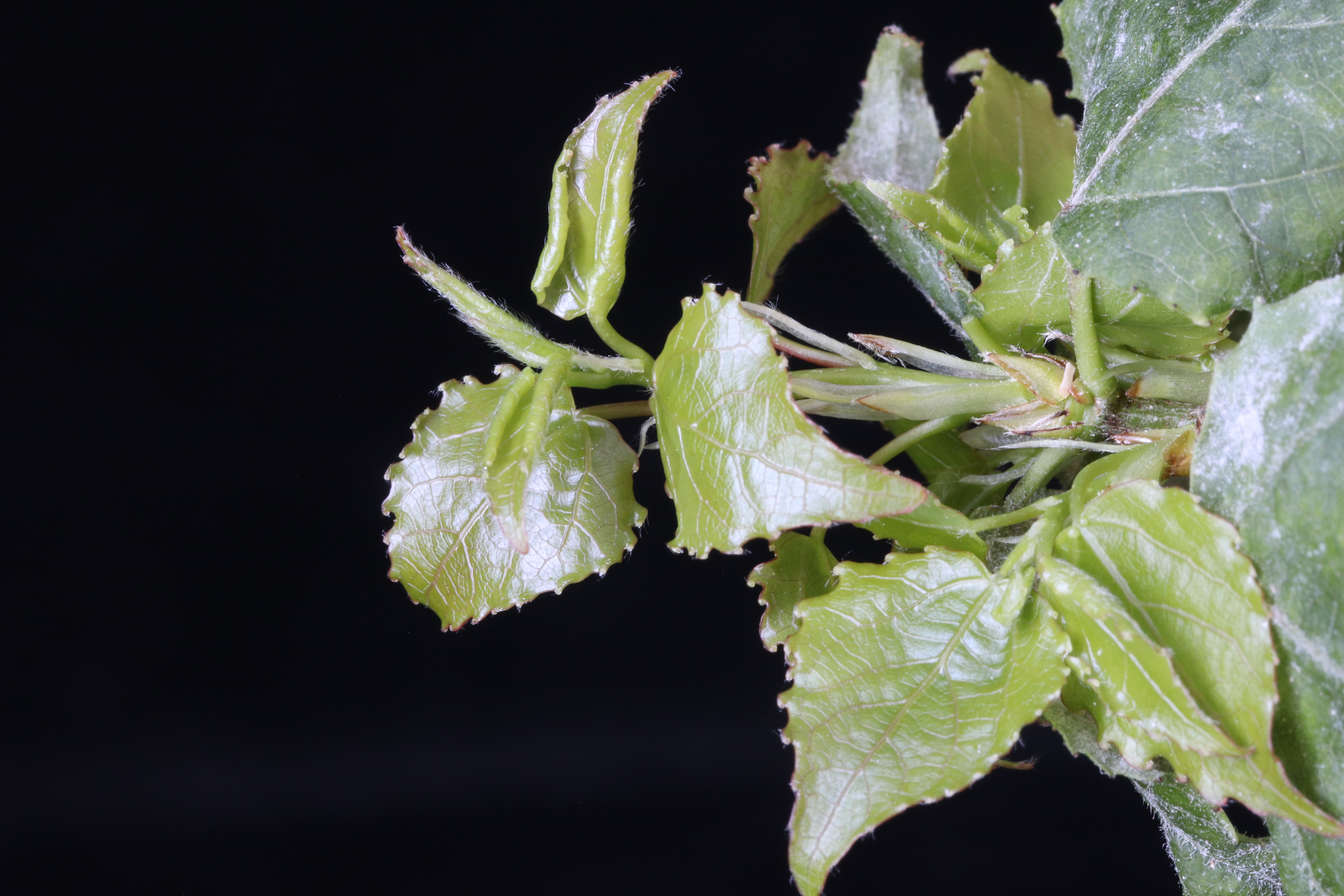

Supplement: Supplementary file 8 — Source data Fig. 6 [file 44318_2024_256_MOESM8_ESM.zip › SD Figure 6/Fig 6A-C/A/4. 6A-LIM1oe-ft1-6.JPG]

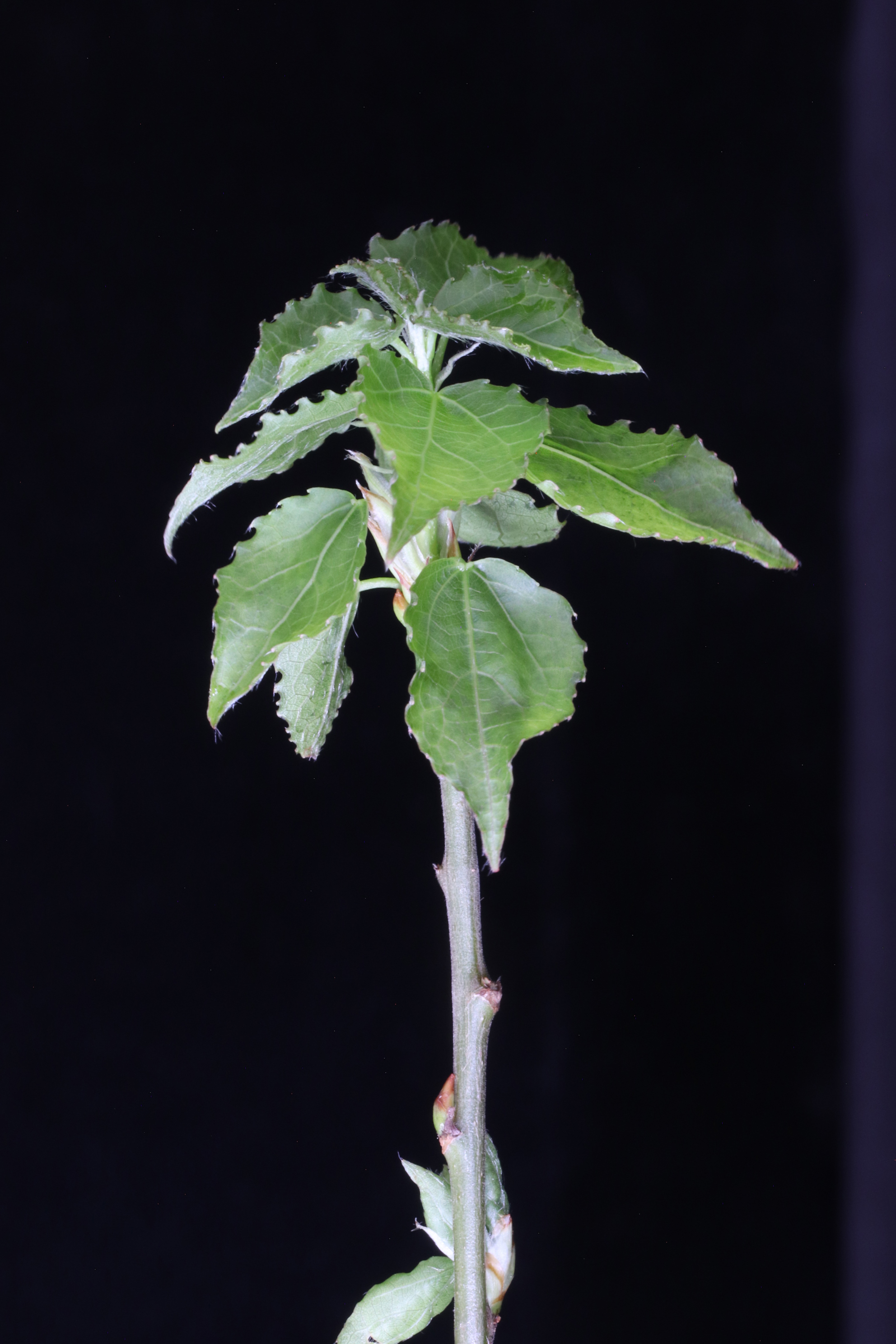

Supplement: Supplementary file 8 — Source data Fig. 6 [file 44318_2024_256_MOESM8_ESM.zip › SD Figure 6/Fig 6A-C/C/2. 6C-Mock_LD.jpg]

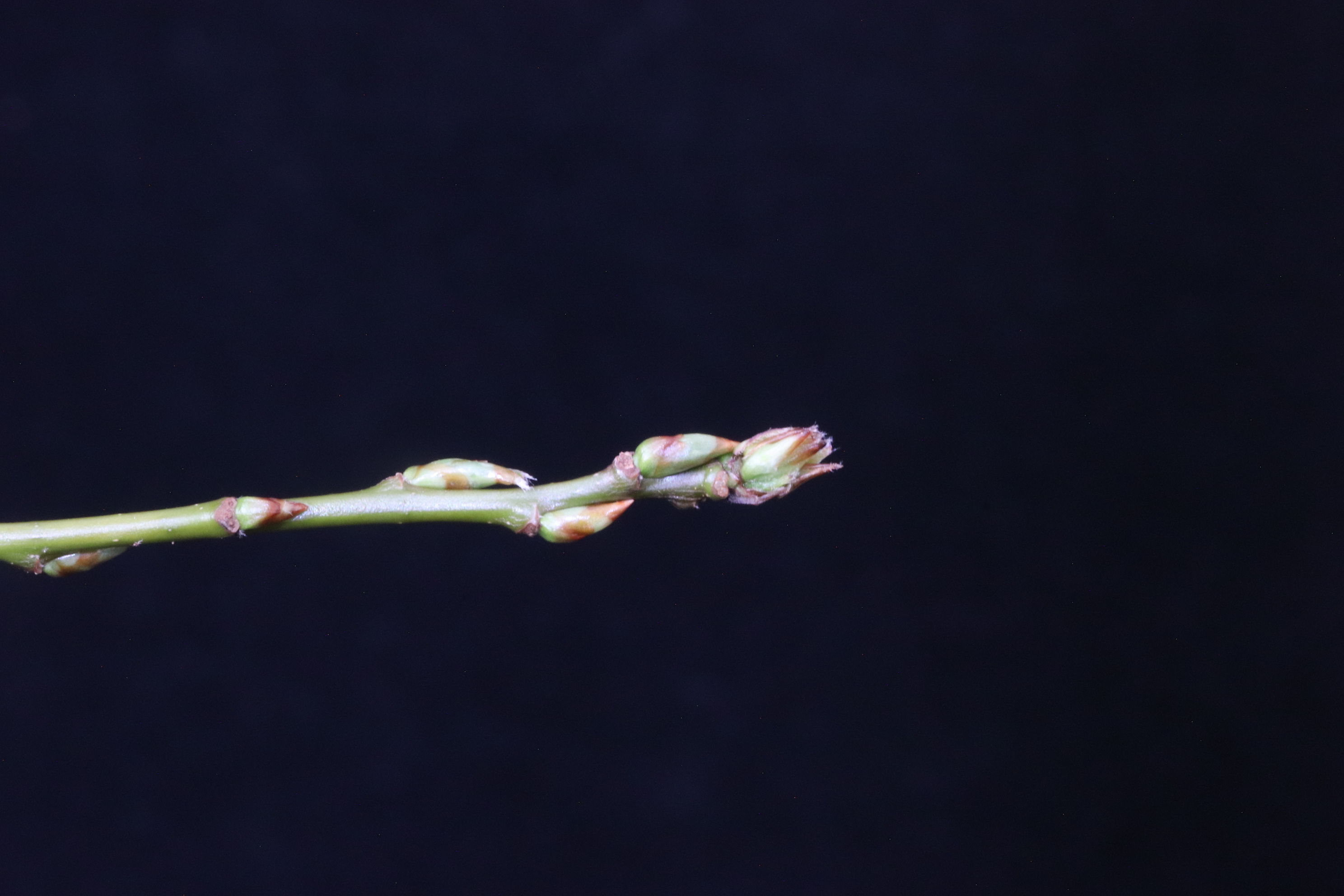

Supplement: Supplementary file 8 — Source data Fig. 6 [file 44318_2024_256_MOESM8_ESM.zip › SD Figure 6/Fig 6A-C/C/4. 6C-PAC_LD.JPG]

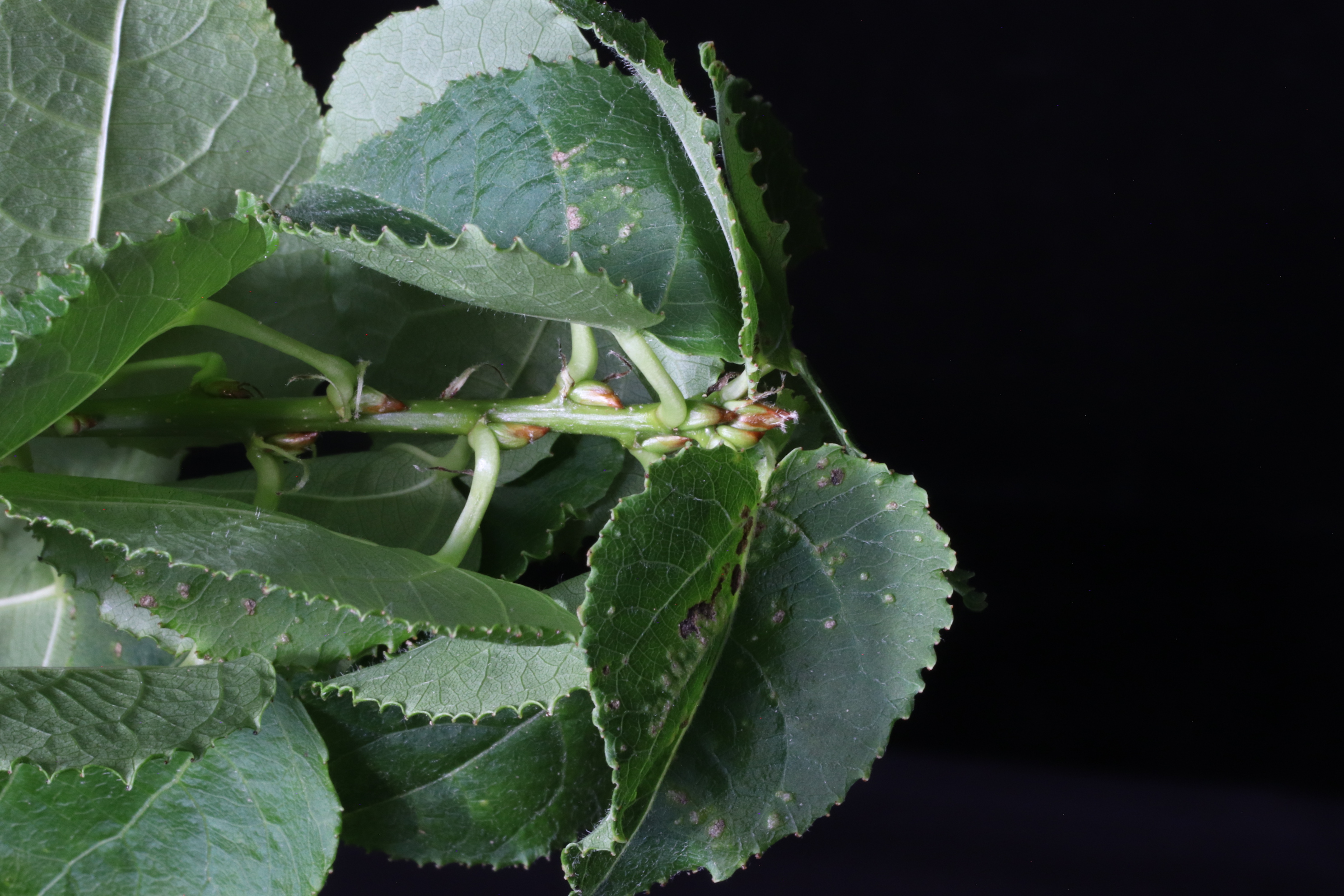

Supplement: Supplementary file 8 — Source data Fig. 6 [file 44318_2024_256_MOESM8_ESM.zip › SD Figure 6/Fig 6A-C/C/3. 6C-PAC_SD.JPG]

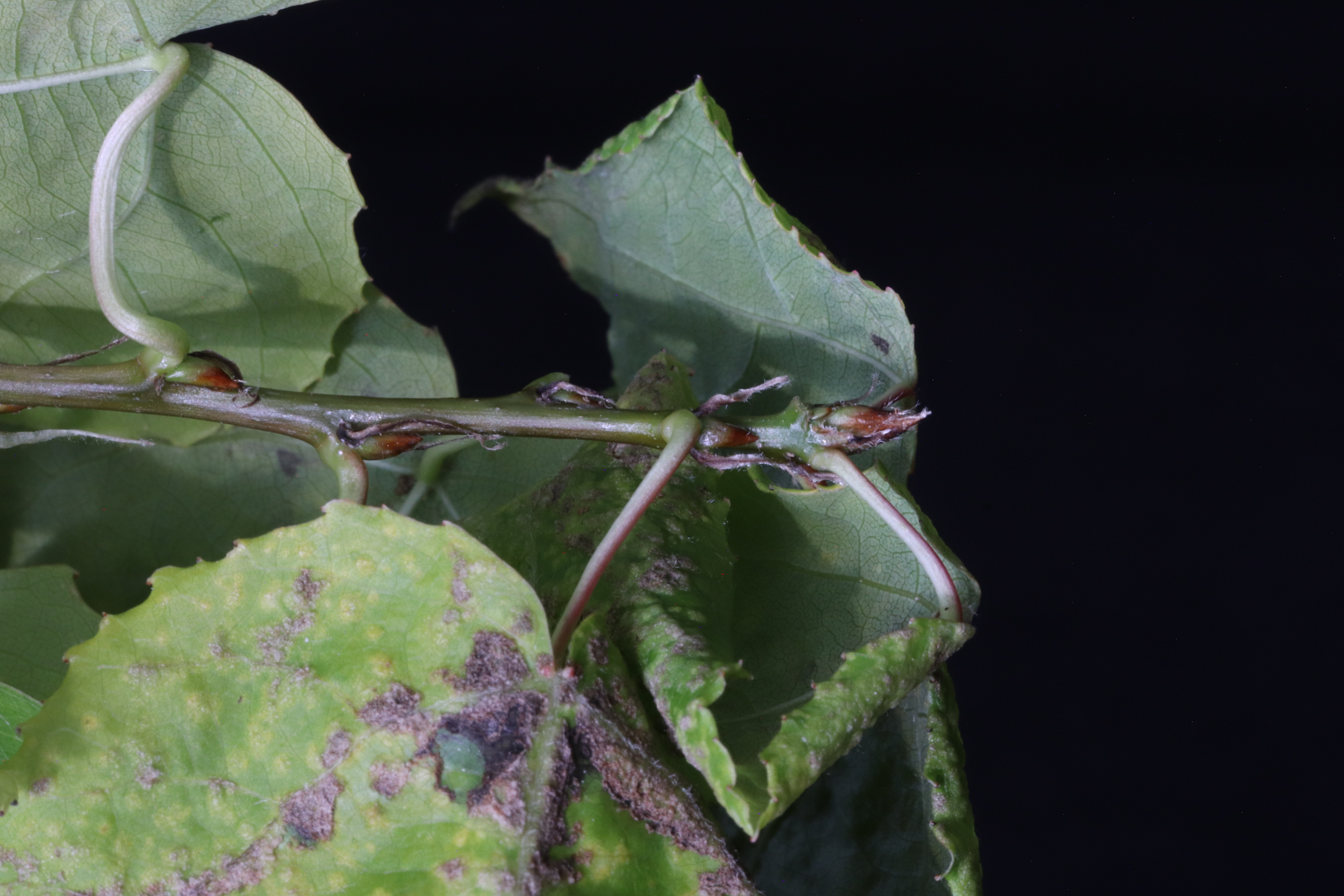

Supplement: Supplementary file 8 — Source data Fig. 6 [file 44318_2024_256_MOESM8_ESM.zip › SD Figure 6/Fig 6A-C/C/1. 6C-Mock_SD.JPG]

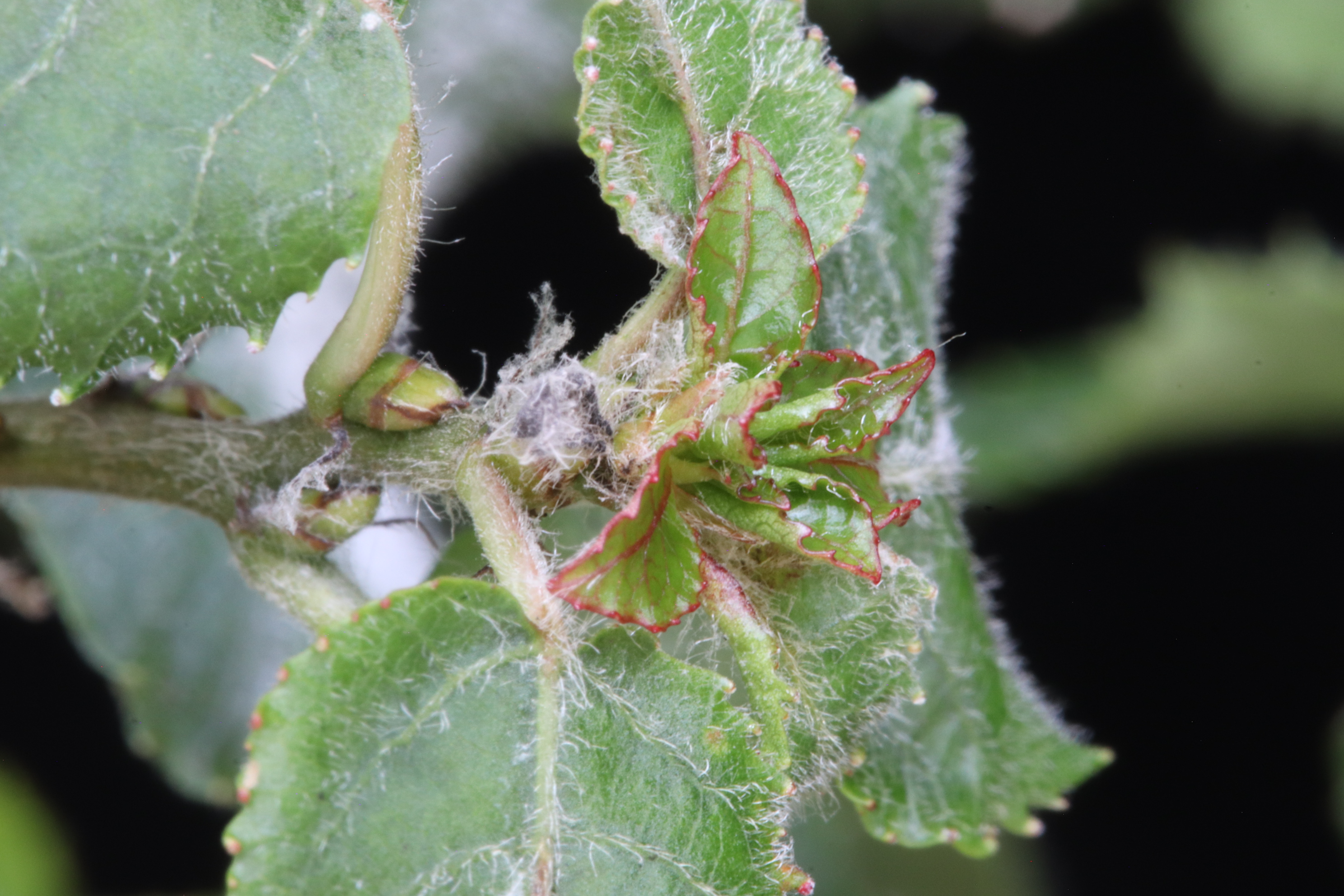

Supplement: Supplementary file 8 — Source data Fig. 6 [file 44318_2024_256_MOESM8_ESM.zip › SD Figure 6/Fig 6A-C/B/4. 6B-LIM1oe-GA2oxoe-6.JPG]

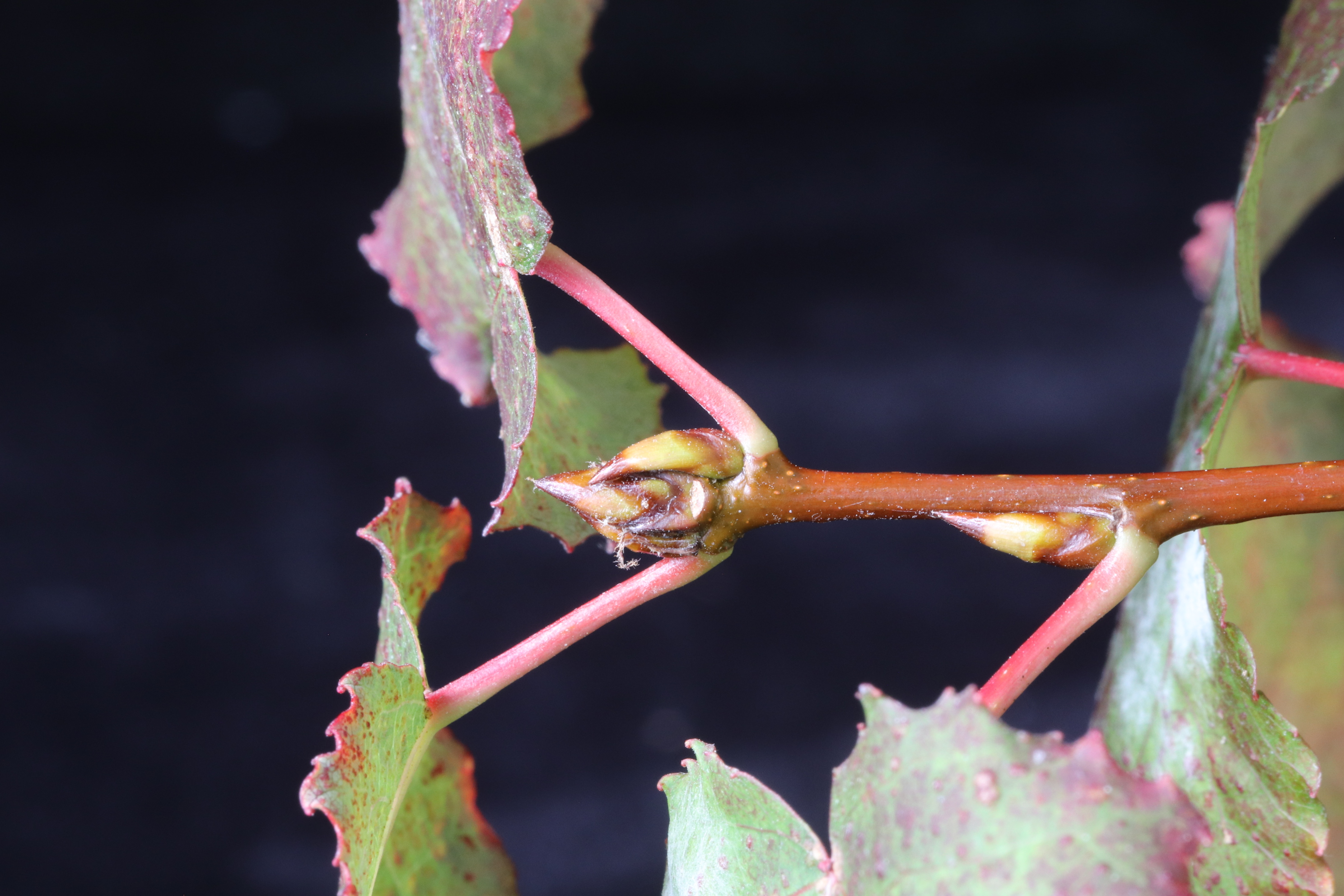

Supplement: Supplementary file 8 — Source data Fig. 6 [file 44318_2024_256_MOESM8_ESM.zip › SD Figure 6/Fig 6A-C/B/1. 6B-WT.JPG]

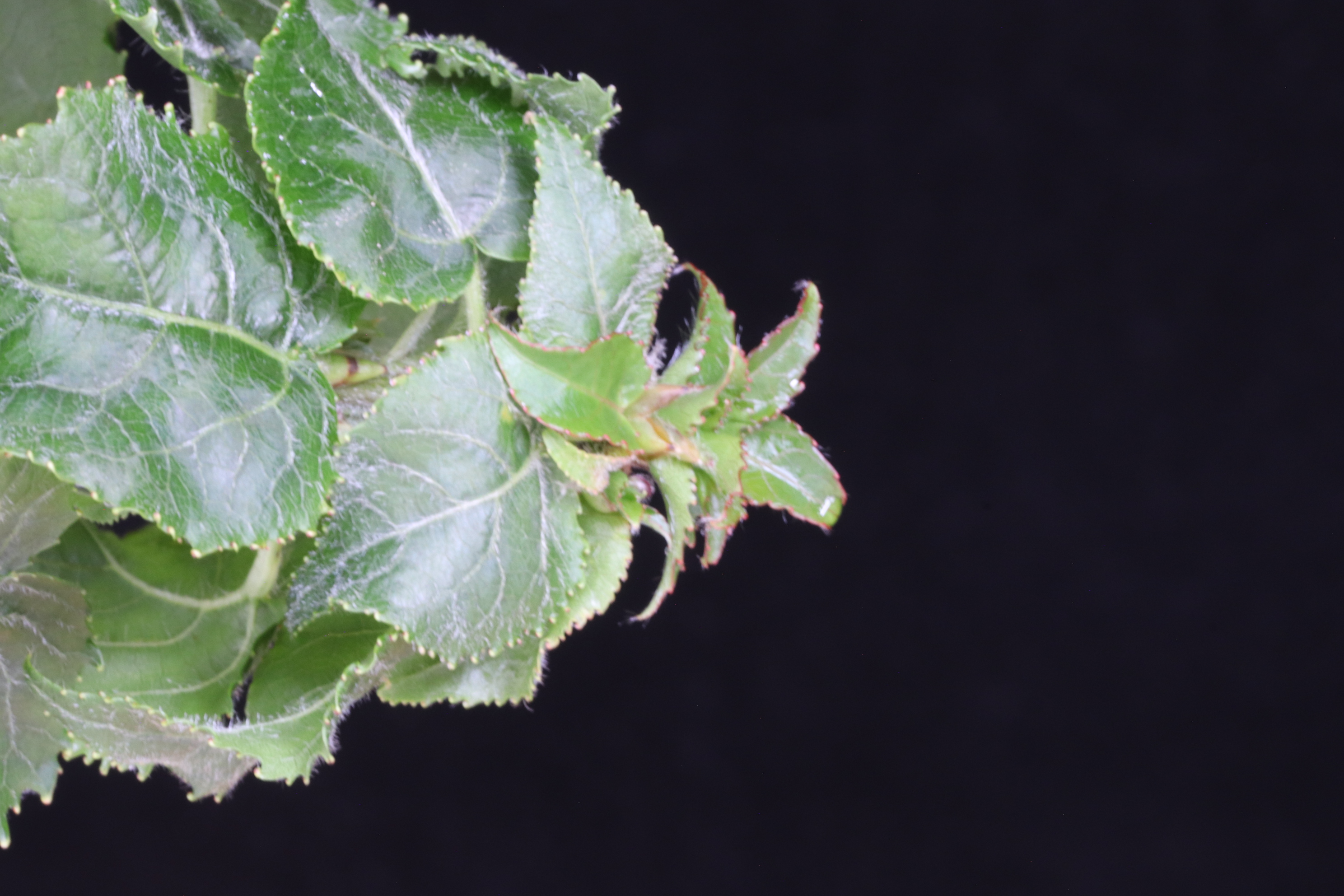

Supplement: Supplementary file 8 — Source data Fig. 6 [file 44318_2024_256_MOESM8_ESM.zip › SD Figure 6/Fig 6A-C/B/3. 6B-LIM1oe-GA2oxoe-2.JPG]

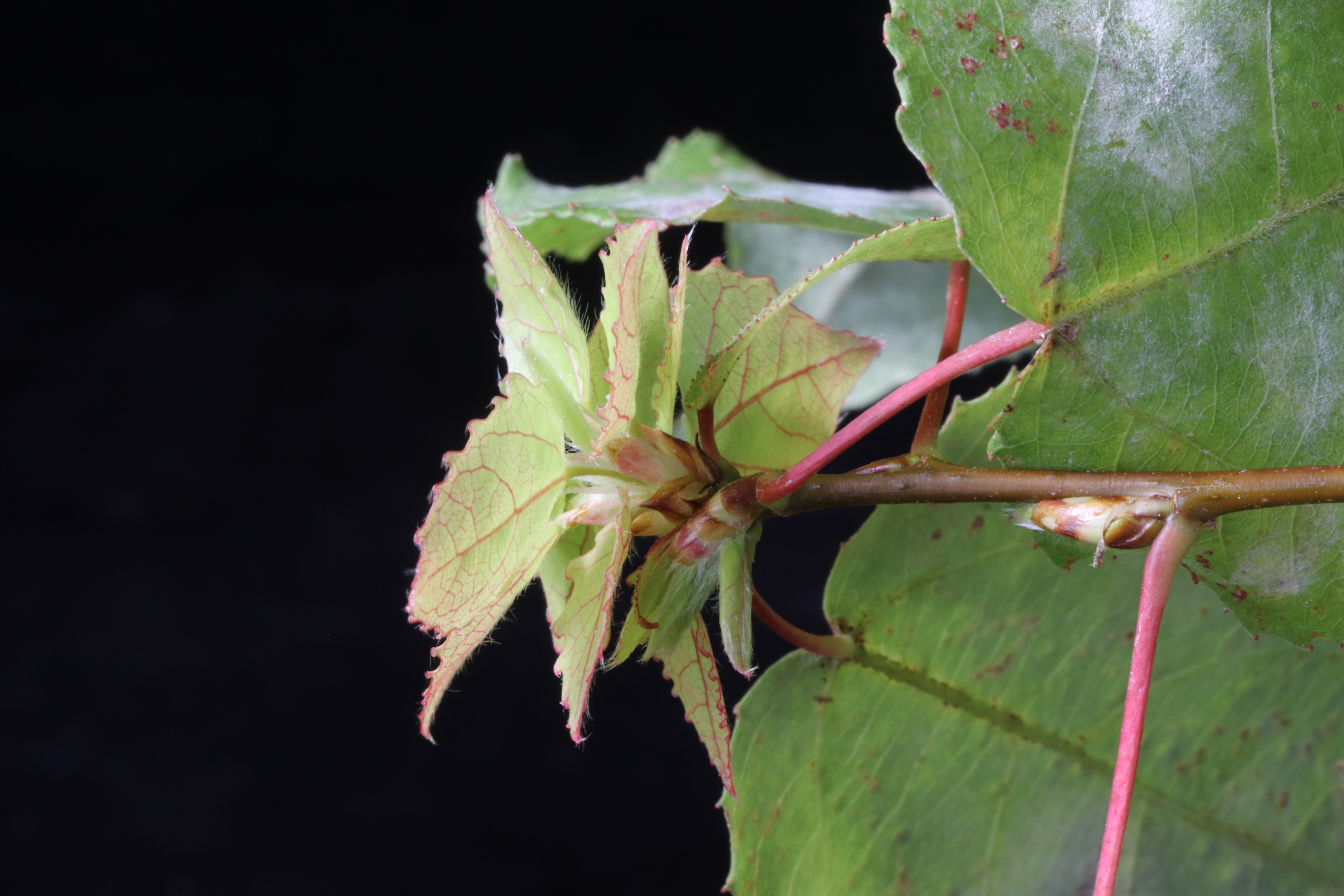

Supplement: Supplementary file 8 — Source data Fig. 6 [file 44318_2024_256_MOESM8_ESM.zip › SD Figure 6/Fig 6A-C/B/2. 6B-LIM1oe.JPG]
