# Supplementary material for: A regulatory module mediating temperature control of cell-cell communication facilitates tree bud dormancy release
Source: EMBO J. 2024 Oct 3;43(23):4. doi: 10.1038/s44318-024-00256-5 (PMC11612439; doi:10.1038/s44318-024-00256-5)
Supplement: Supplementary file 10 — Expanded View Figures [file 44318_2024_256_MOESM10_ESM.pdf]

Expanded View Figures

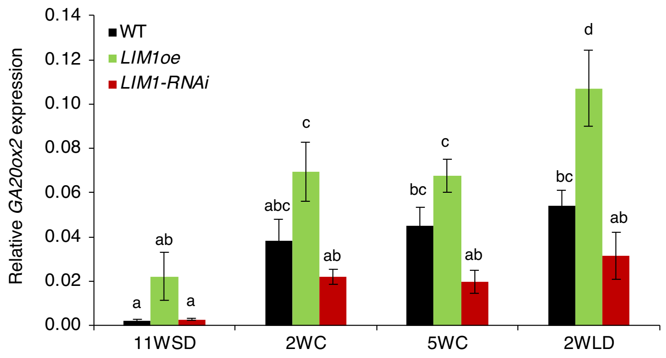

Figure EV1. LIM1 is a positive regulator of gibberellic acid (GA) pathway.

Relative expression of *GA20ox2* in buds of wild-type (WT), *LIM1oe*, and *LIM1-RNAi* plants at 11 weeks short days (11WSD), 2 (2WC), and 5 weeks of cold (5WC) and after 2 weeks of warm temperature (20 °C) in long days (2WLD). The expression values are relative to the reference gene UBQ and the average of three biological replicates. Error bars indicate standard error mean (±SEM). Different letters over the bars indicate statistically significant differences at $P < 0.05$ by one-way ANOVA Duncan's test.

© The Author(s)

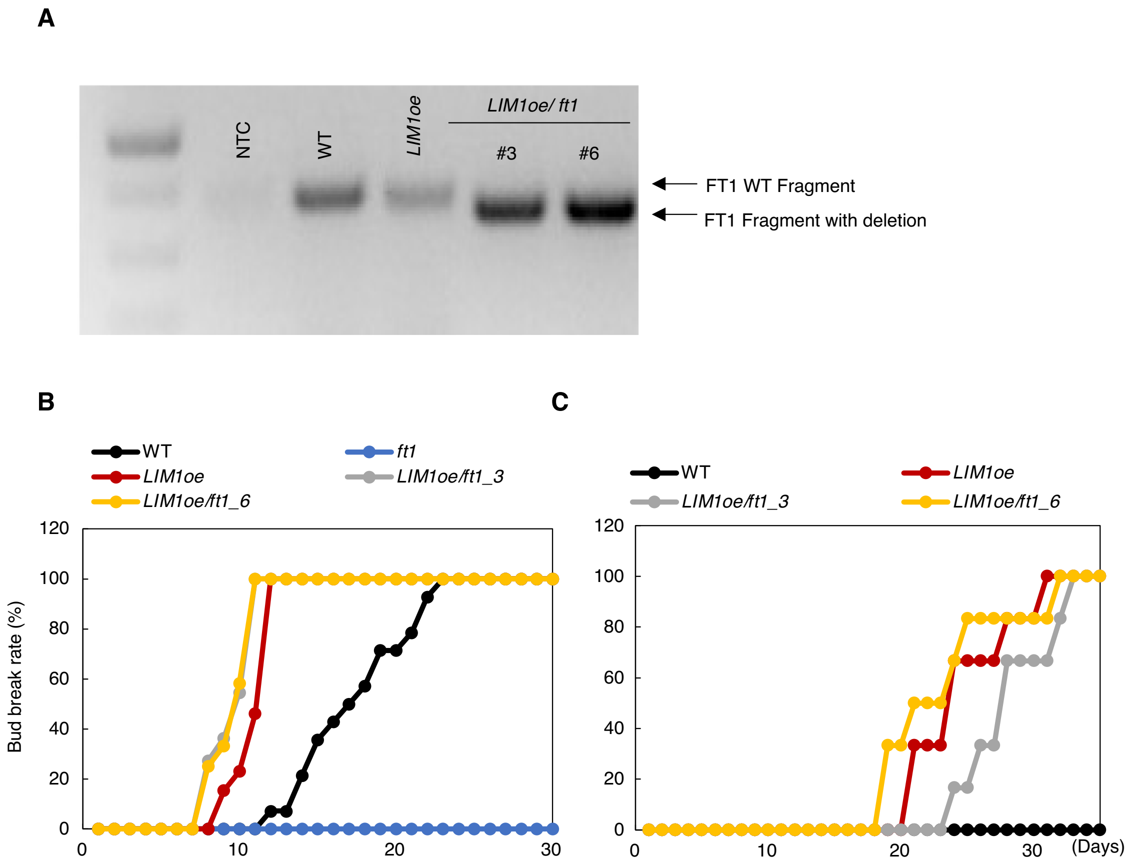

Figure EV2. LIM1 and FT1 function in a partially redundant manner.

(**A**) Screening of *ft1* knockout in *LIM1oe/ft1* double transgenic lines. The polymerase chain reaction (PCR) result showing the detection of CRISPR/Cas9-mediated FT1 deletion in *LIM1oe/ft1* lines by agarose gel electrophoresis. PCR products *c.* 400 bp size corresponded to the wild-type (WT) variant, and the FT1 amplicon in *LIM1oe/ft1* lines of *c.* 350 bp indicated the internal deletion. NTC stands for no template control (**B**) The bud break rate (%) of WT, *LIM1oe,* and *LIM1oe/ft1* (lines 3 and 6) plants grown under SD for 11 weeks, then treated with cold (4 °C) for 5 weeks followed by transfer to LD for bud burst analysis. (**C**) The bud break rate (%) of WT, *LIM1oe,* and *LIM1oe/ft1* plants moved directly from 11 weeks of short-day conditions (SD) to long-day conditions (LD), without a cold treatment, corresponds to Fig. 6A). The experiments (**B** and **C**) were repeated at least twice with similar results, and the bud-break rate (%) is shown with data from 7 to 10 plants from each line.

 © The Author(s)

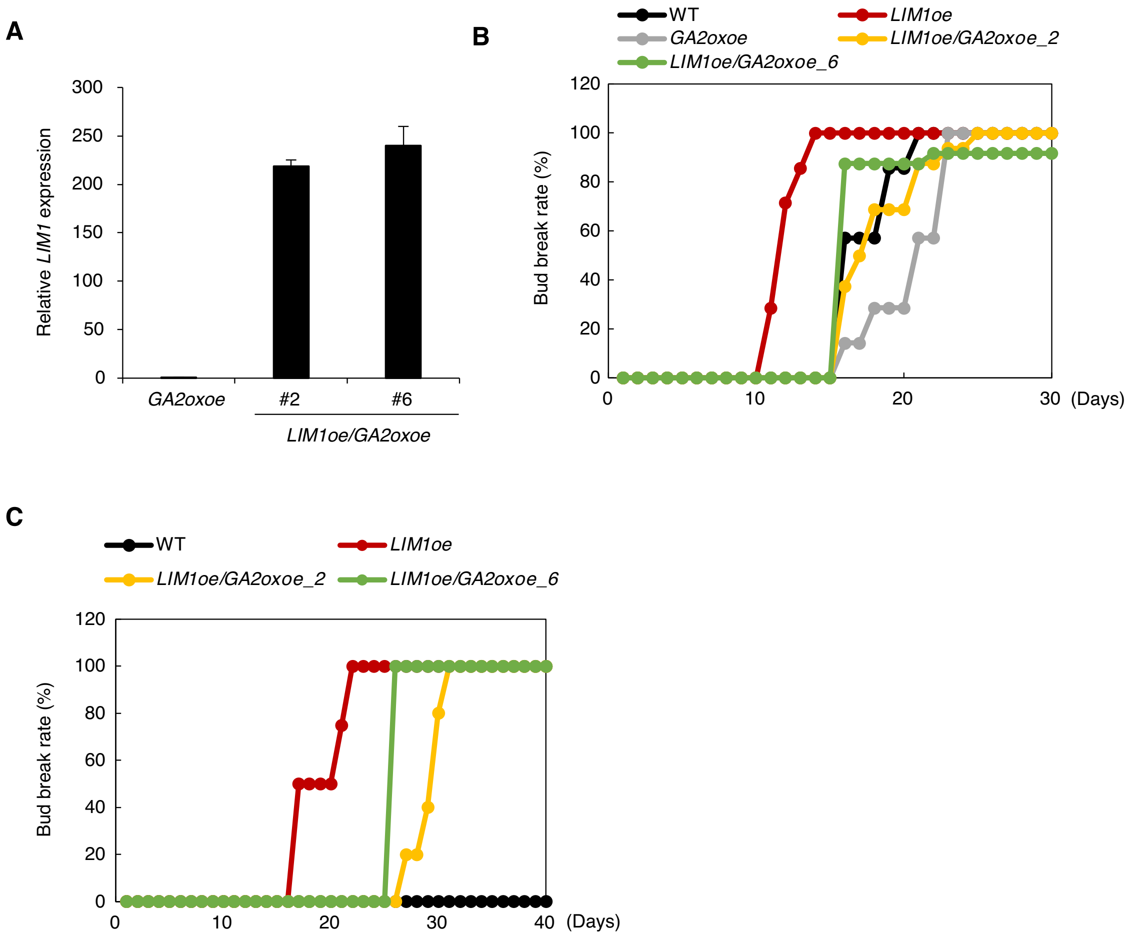

Figure EV3. LIM1 and FT1 redundantly converge on the gibberellic acid (GA) pathway.

(**A**) *LIM1* expression in *LIM1oe/GA2oxoe* lines. See Fig. 6 (**B**). Expression values shown are normalized to the reference gene UBQ and are averages of three biological replicates (±SEM). (**B**) Bud break phenotypes of WT, *LIM1oe, GA2oxoe* and *LIM1oe/GA2oxoe* plants grown under SD for 11 weeks, then treated with cold (4 °C) for 5 weeks followed by transfer to LD for bud burst analysis. (**C**) Bud-break rate (%) of wild-type (WT), *LIM1oe* and *LIM1oe/GA2oxoe* line plants moved directly from 11 weeks of short-day conditions (SD) to long-day conditions (LD) without cold treatment, corresponding to Fig. 6B. The experiments (**B** and **C**) were repeated at least twice with similar results, and the bud-break rate (%) is shown with data from 7 to 10 plants from each line.

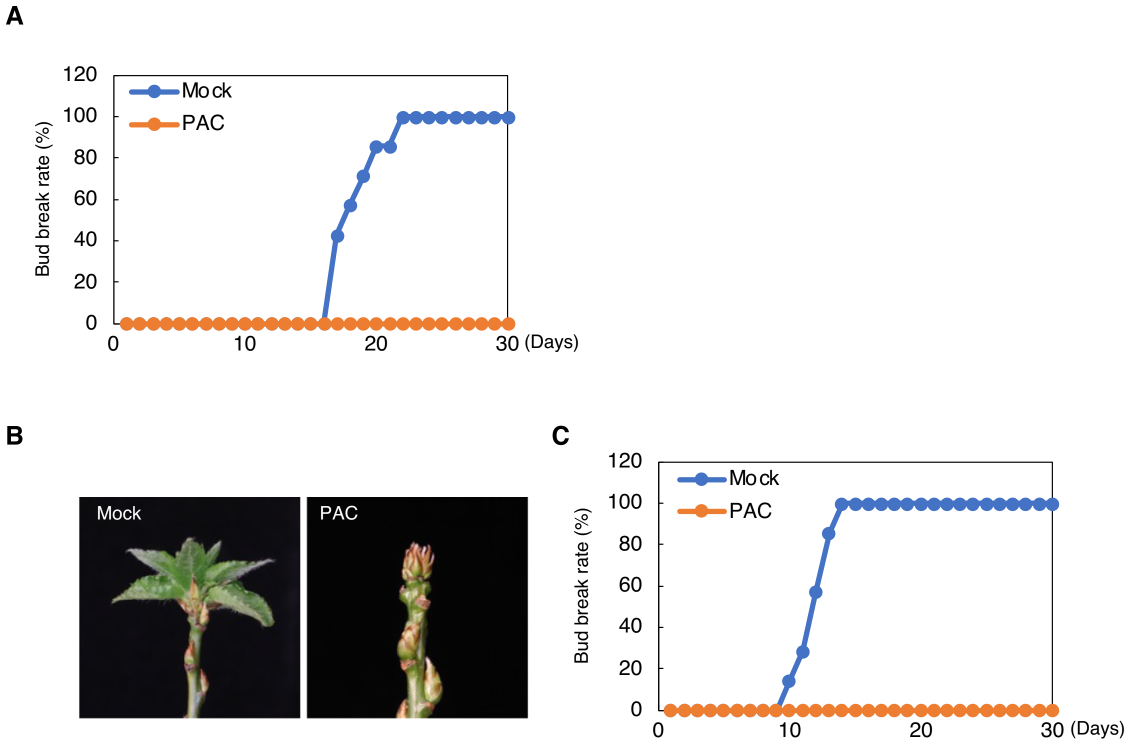

Figure EV4. GA is the downstream target of *LIM1* and *FT1*.

(**A**) Bud-break rate (%) of *LIM1oe/FT1crispr* plants treated with Mock and paclobutrazol (PAC) moved directly from 11 weeks of short-day (SD) to long-day conditions (LD) without cold treatment, corresponding to Fig. 6C. (**B**, **C**) Bud break phenotypes of *LIM1oe/FT1crispr* plants treated with Mock and paclobutrazol (PAC) under long-day conditions (LD) after cold treatment. Bud break phenotyping were repeated at least twice with similar results, and the bud-break rate (%) is shown with data from 7 plants.

 © The Author(s)
